# Structure-Based Optimization of Inhibitors of the Aspartic Protease Endothiapepsin

**DOI:** 10.3390/ijms160819184

**Published:** 2015-08-14

**Authors:** Alwin M. Hartman, Milon Mondal, Nedyalka Radeva, Gerhard Klebe, Anna K. H. Hirsch

**Affiliations:** 1 Stratingh Institute for Chemistry, University of Groningen, Nijenborgh 7, 9747 AG Groningen, The Netherlands; E-Mails: a.m.hartman@student.rug.nl (A.M.H.); m.mondal@rug.nl (M.M.); 2 Institute of Pharmaceutical Chemistry, Philipps-University Marburg, Marbacher Weg 6, 35032 Marburg, Germany; E-Mails: radeva.neli@uni-marburg.de (N.R.); Klebe@staff.uni-marburg.de (G.K.)

**Keywords:** inhibitors, aspartic protease endothiapepsin, structure-based drug design, molecular recognition

## Abstract

Aspartic proteases are a class of enzymes that play a causative role in numerous diseases such as malaria (plasmepsins), Alzheimer’s disease (β-secretase), fungal infections (secreted aspartic proteases), and hypertension (renin). We have chosen endothiapepsin as a model enzyme of this class of enzymes, for the design, preparation and biochemical evaluation of a new series of inhibitors of endothiapepsin. Here, we have optimized a hit, identified by *de novo* structure-based drug design (SBDD) and DCC, by using structure-based design approaches focusing on the optimization of an amide–π interaction. Biochemical results are in agreement with SBDD. These results will provide useful insights for future structure-based optimization of inhibitors for the real drug targets as well as insights into molecular recognition.

## 1. Introduction

Aspartic proteases are a class of enzymes widely found in fungi, plants, vertebrates, as well as, e.g., in HIV retro-viruses. These enzymes are involved in several diseases, such as Alzheimer’s disease, amyloid disease, malaria, fungal infections, hypertension and AIDS [1]. In HIV, the aspartic protease has an essential role in maturation of the HIV virus, making it a validated target for the treatment of AIDS. In eukaryotes, the aspartic protease renin plays a role in hypertensive action, cathepsin D in tumorigenesis, plasmepsins in the degradation of human hemoglobin, which is required by *Plasmodium falciparum*, the causative agent of malaria and pepsin in the hydrolysis of acid-denatured proteins. Therefore, the enzymes of this class of aspartic proteases are considered as a rich source of therapeutic targets.

Endothiapepsin belongs to the family of pepsin-like aspartic proteases. Owing to the high degree of similarity, it has served as the model enzyme for the identification of inhibitors of renin [2] and β-secretase [3] and has been used to elucidate the mechanism of aspartic proteases [4,5,6]. It is a robust enzyme, active at room temperature for more than 20 days, available in large quantities. It crystallizes easily and provides very well-diffracting crystals, making this enzyme an appropriate model enzyme for aspartic proteases [7]. Pepsin-like aspartic proteases are active as monomers, which comprise two similar domains, each of which contributes an aspartic acid residue to the catalytic dyad (D35 and D219 for endothiapepsin) that hydrolyzes the substrate’s peptide bond utilizing a catalytic water molecule.

In the present study, we have used structure-based drug design (SBDD) [8,9,10], to optimize an acylhydrazone-based inhibitor of endothiapepsin.

## 2. Results and Discussion

**Figure 1:**
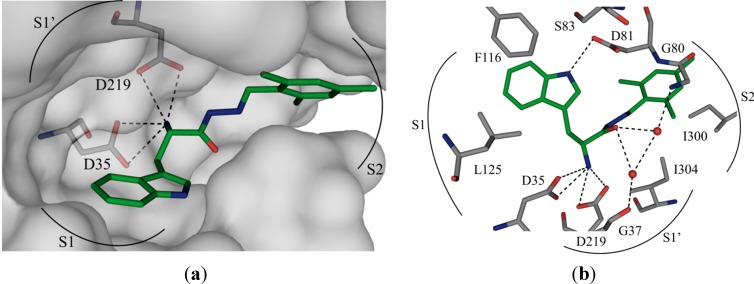
(**a**) X-ray crystal structure of endothiapepsin co-crystallized with **1**; (**b**) full binding mode of **1** in the active site. Color code: inhibitor skeleton: C: green, N: blue, O: red; enzyme skeleton: C: gray; water molecules: red sphere. H bonds below 3.2 Å are shown as black, dashed lines (PDB code: 4KUP) [7].

We chose the acylhydrazone-based hit **1** as a starting point. We had previously identified hit **1** from a dynamic combinatorial library of acylhydrazones using the novel combination of *de novo* SBDD and dynamic combinatorial chemistry (DCC) [7]. Our hit **1** displays an IC_50_ value of 12.8 μM against endothiapepsin and binds in the active site of the enzyme (Figure 1a). **1** forms four charged H bonds with the catalytic dyad (D35 and D219) through its *α*-C amino group. The carbonyl oxygen atom of the acylhydrazone linker accepts two H bonds from two co-crystallized water molecules (Figure 1b). Furthermore, the NH of the indolyl moiety donates an H bond to the carboxylate group of the residue D81. **1** addresses the S1 pocket of endothiapepsin with its indolyl moiety and benefits from offset π–π stacking and CH–π interactions with F116 and L125, respectively. Furthermore, **1** experiences several hydrophobic interactions with I300 and I304 in the S2 pocket of endothiapepsin. Moreover, the mesityl group is involved in an amide–π interaction with the peptide bond connecting G80 and D81.

**Scheme 1:**
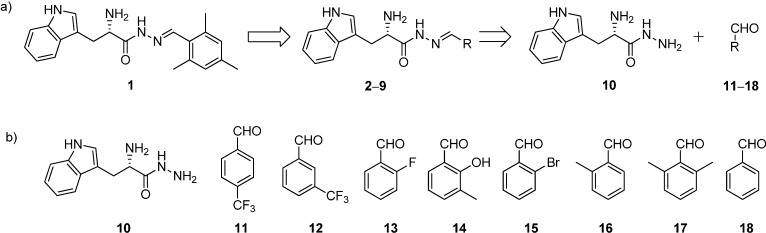
(**a**) Structures and retrosynthetic analysis of designed acylhydrazone inhibitors **2**–**9** starting from hit **1**; (**b**) structures of hydrazide **10** and the aldehydes **11**–**18**.

We focused our efforts on optimizing the amide–π interaction [11]. Generally speaking, electron-deficient rings, which have their dipole moment aligned in an antiparallel manner with the peptide bond should strengthen the amide–π stacking interaction. Furthermore, an increase in dipole moment should also increase the strength of the amide–π stacking interaction [11]. With these considerations in mind, we embarked on the optimization of this interaction by varying the aromatic ring derived from the aldehyde building block. Based on molecular modeling using the software Moloc [12] and the FlexX docking module in the LeadIT suite [13] as well as commercial availability, we have designed and selected a library of eight acylhydrazone-based potential hits **2**–**9** (Scheme 1a). We have introduced various electron-withdrawing group such as –F, –CF_3_, and –Br as well as the electron-donating group –OH to investigate the effect of varying the electron density in the aromatic ring to improve the amide–π interaction between the aromatic ring and the peptide bond connecting G80 and D81 as well as to improve the offset π–π stacking, CH–π interaction and several hydrophobic interactions. The binding modes of all designed acylhydrazone analogues are shown in Figure S1 in the Supplementary information. Substituting the methyl by a trifluoromethyl group might have several beneficial effects. One of the most common reasons to incorporate fluorine in an inhibitor is that the rate of oxidative metabolism is reduced. Furthermore, the fluorine atom (van der Waals radius, 1.47 Å) is able to mimic a hydrogen atom (1.20 Å) or a hydroxyl group (1.40 Å) in a bioactive compound because it is of comparable size. The stability towards biological oxidation is strongly determined by the bond energies and heats of formation of F–O relative to C–O and H–O bonds. The stronger C–F bond, compared to C–H, does not influence the biological oxidative stability, due to the fact that homolysis of C–H or C–F bonds is not involved [14]. Due to the potentially higher stability towards oxidative metabolism, replacing the mesityl by a trifluoromethylphenyl substituent should decrease the risk for toxicity. Moreover, the presence of fluorine atoms can enhance the lipophilicity and therefore the *in vivo* uptake and transport of biologically active compounds.

**Figure 2:**
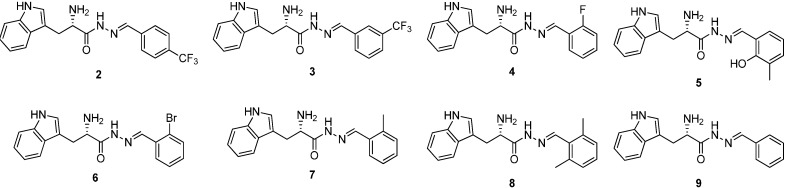
Structures of a series of acylhydrazone-based inhibitors **2**–**9**.

**Scheme 2:**
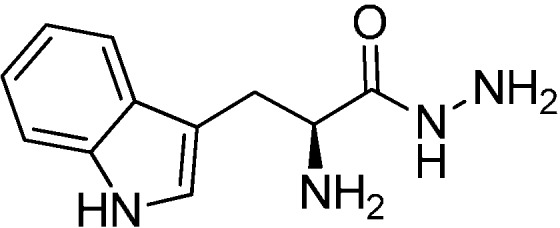
Structure of (*S*)-2-amino-3-(1*H*-indol-3-yl)propanehydrazide **10**.

**Scheme 3:**
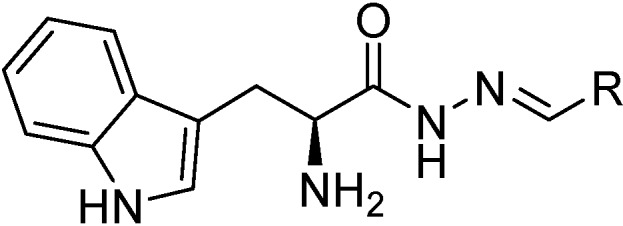
General structure of acylhydrazones **2**–**9**.

All the acylhydrazone derivatives can be synthesized by treating l-tryptophan hydrazide (**10**) with eight aldehydes **11**–**18** to afford the corresponding acylhydrazones **2**–**9** (Scheme 1). Whereas, all the aldehydes are commercially available, we have synthesized the hydrazide **10** starting from l-tryptophan methyl ester hydrochloride (**19**) by treatment with hydrazine monohydrate as reported previously (Scheme 2 and Scheme S1a in supplementary information) [7]. We accessed all acylhydrazones **2**–**9** (Figure 2) by reacting hydrazide **10** with the individual aldehydes **11**–**18** and isolated the acylhydrazones as mixtures of *E* and *Z* isomers in 30%–50% yield (Scheme 3, Schemes S1b and S2–S9, Figures S2–S28 in Supplementary information) [7].

To determine their inhibitory potency against endothiapepsin, we subjected these acylhydrazone derivatives to a fluorescence-based enzymatic inhibition assay, adapted from the HIV protease assay [15]. All eight acylhydrazones indeed showed inhibition of endothiapepsin with IC_50_ values in the range of 7–59 µM except for **9**, which showed an IC_50_ value of 244 μM. The most potent inhibitor **2** displays an IC_50_ value of 7.0 μM. The experimental Gibbs free energies of binding (Δ*G*_EXPT_ (**2**) = −31.3 kJ·mol^−1^) and ligand efficiencies (*LE* (**2**) = 0.28), obtained from the IC_50_ values using the Cheng–Prusoff equation [16], correlate with the calculated value using the scoring function HYDE in the LeadIT suite Δ*G*_HYDE_ (**2**) = −32.0 kJ·mol^−1^ [17,18]. This correlation is also valid for other acylhydrazones, except for **3**, **6** and **7** (Table 1).

**Table 1:** The IC_50_ values, ligand efficiency (*LE*), calculated and experimental Gibbs free energy of binding (Δ*G*) of acylhydrazone inhibitors.

Inhibitors	*E*:*Z* ^a^	IC_50_ (μM) ^b^	Δ*G*_expt_ (kJ·mol^−1^) ^c^	*LE*	Δ*G*_HYDE_ (kJ·mol^−1^) ^d^
**1**	57:43	12.8 ± 0.4	−30.0	0.27	−32
**2**	64:36	7.0 ± 0.5	−31.3	0.28	−32
**8**	45:55	30.0 ± 5.0	−27.7	0.26	−32
**5**	93:7	36.0 ± 11.0	−27.2	0.26	−30
**4**	58:42	38.0 ± 7.0	−27.1	0.27	−31
**9**	60:40	49.0 ± 2.0	−26.4	0.27	−31
**6**	48:52	54.5 ± 0.5	−26.2	0.26	−33
**7**	60:40	59.0 ± 4.0	−26.0	0.26	−38
**3**	38:62	244.0 ± 32.0	−22.5	0.20	−33

^a^ *E*/*Z* ratios were calculated based on integration of the peak corresponding to the imine-type proton in the ^1^H NMR spectrum; ^b^ 26 experiments were performed and only six experiments were considered to calculate the initial slope (*n* = 6), 11 different concentrations of inhibitor were used starting at 1 mM; each experiment was carried out in duplicate and the errors are given in standard deviations (SD); ^c^ The Gibbs free energy of binding (Δ*G*; derived from the experimentally determined IC_50_ values); ^d^ Values indicate the calculated Gibbs free energy of binding (Δ*G*_HYDE_; calculated by the HYDE scoring function in the LeadIT suite).

**Figure 3:**
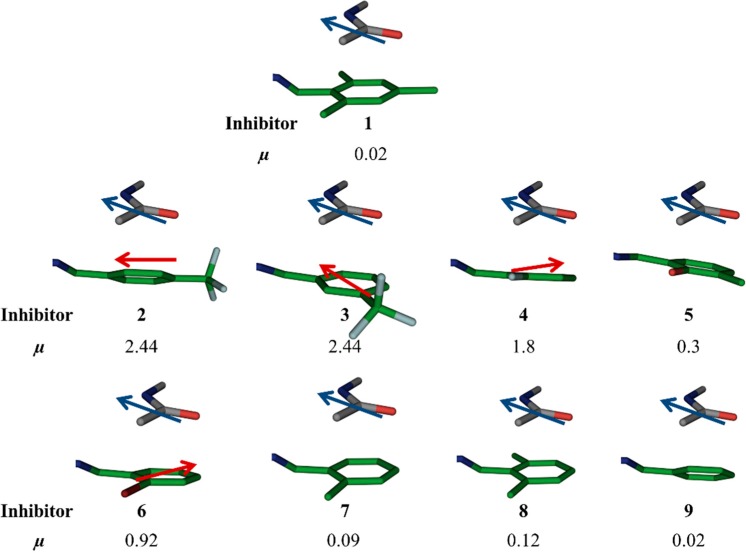
Moloc-generated dipole moments (µ) of aromatic rings of the original hit **1** and designed acylhdrazone inhibitors **2**–**9**.

**Figure 4:**
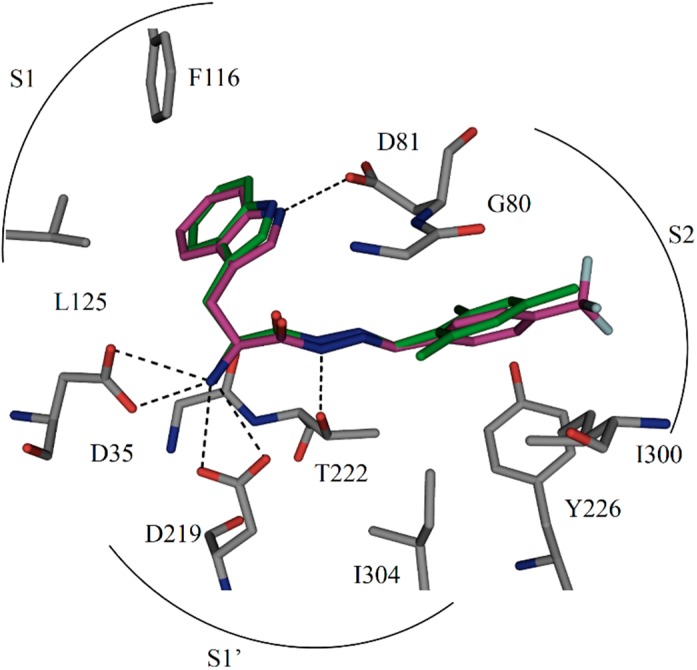
Comparison of the binding mode of crystal structure of **1** and modeled structure of **2** in the active site of endothiapepsin. Color code: inhibitor skeleton: C: green, purple, N: blue, O: red, F: light cyan; enzyme skeleton: C: gray. H bonds below 3.2 Å are shown as black dashed lines (PDB code: 4KUP) [7].

When the *para*-methyl group of the mesityl substituent of **1** is removed, the IC_50_ value increases to 30.0 μM (**8**), which suggests that the *para*-methyl group was involved in lipophilic interactions with I300. Removal of a second methyl group (**7**) leads to another two-fold increase in IC_50_ to 59.0 µM. When the last methyl group (*ortho*) is removed, *i.e.*, the unsubstituted phenyl derivative **9**, the IC_50_ value (49.0 µM) is in the same range as for the aromatic ring with one methyl group (**7**), which indicates that one of the *ortho* methyl groups was not involved in any lipophilic interactions. Upon introduction of a trifluoromethyl group in the *para* position of the phenyl ring (**2**), the IC_50_ value, decreases two-fold to 7.0 µM with respect to the initial hit **1**, which could be due to the better liphophilic interactions and stronger amide–π interactions. However, the IC_50_ value increases to 244.0 µM in case of the *meta*-trifluoromethyl-substituted derivative **3**. This observation suggests that the *para* trifluoromethyl group is involved in more lipophilic interaction than the *meta* trifluoromethyl group. In case of *ortho*-fluorophenyl (**4**) and -bromophenyl (**6**) substituents, the IC_50_ values are in the same range as the unsubstituted phenyl derivative (**9**), which indicates that fluoro and bromo substituents in the *ortho* position do not have a strong influence on the binding event. Introduction of a hydroxyl group in the *ortho* position along with a methyl group in the *meta* position (**5**) leads to an IC_50_ value of 36.0 µM, which suggests that the hydroxyl group in the *ortho* position might be involved in H bonding. Therefore, the highest potency observed for **2** might be ascribed to the strongly electron-withdrawing properties of the trifluoromethyl substituent in *para* position, which makes the aromatic ring electron-deficient, which, in turn, should strengthen the amide–π interaction. The alignment of dipole moments of the amide bond and the aromatic ring is not ideal (*i.e.*, antiparallel) as observed from dipole-moment calculations using molecular modeling using the software Moloc (Figure 3) [12]. Lipophilic interactions between the fluorine atoms and nearby lipophilic residue I300 certainly also contribute to the potency. A superimposition of hit **1** and the modeled binding pose of the most potent inhibitor **2** is shown in Figure 4. Moreover, because of the higher stability towards oxidative metabolism, trifluoromethylphenyl should reduce the risk for toxicity compared to mesityl. At the same time, the presence of fluorine atoms can enhance the lipophilicity and consequently the *in vivo* uptake and transport of biologically active compounds.

## 3. Experimental Sections

### 3.1. General Experimental Details

Starting materials and reagents were purchased from Aldrich, (Zwijndrecht, The Netherlands) or Acros (Geel, Belgium). Yields refer to analytically pure compounds and have not been optimized. All solvents were reagent-grade and if necessary, SPS-grade. Column chromatography was performed on silica gel (Silicycle^®^ Silia*Sep*^™^ 40–63 μM 60 Å). TLC was performed with silica gel 60/Kieselguhr F254. Solvents used for the column chromatography were dichloromethane and methanol. ^1^H, ^13^C and ^19^F spectra were recorded at 400 MHz on a Varian AMX400 spectrometer (Agilent Technologies, Santa Clara, CA, USA) (400 MHz for ^1^H, 101 MHz for ^13^C and 376 MHz for ^19^F) at 25 °C. Acylhydrazone NMR spectra consist of both *E* and *Z* isomers.

Chemical shifts (δ) are reported relative to the residual solvent peak. Splitting patterns are indicated as (s) singlet, (d) doublet, (t) triplet, (q) quarted, (m) multiplet, (br) broad. The coupling constants (*J*) are given in Hz. NMR spectra consist of signals from both *E* and *Z* isomers. High-resolution mass spectra were recorded with an FTMS orbitrap (Thermo Fisher Scientific, Waltham, MA, USA) mass spectrometer. FT-IR were measured on a PerkinElmer FT-IR spectrometer, Waltham, MA, USA. Melting points were measured on a Stuart^®^ SMP11 50 W melting point apparatus.

HPLC conditions: column, XTerra^®^ MS C18 3.5 µm, 3.0 × 150 mm; flow rate 0.5 mL·min^−1^; wavelength, 254 nm; temperature, 23 °C; gradient, H_2_O/MeCN (0.1% TFA) from 95% to 5% over 20 min, then at 5% for 2 min. Optical rotations were measured in MeOH on a Schmidt & Haensch polarimeter (Polartronic MH8, Berlin, Germany) with a 10 cm cell (*c* given in g/100 mL).

### 3.2. (S)-2-Amino-3-(1H-indol-3-yl)propanehydrazide (**10**)

Hydrazine monohydrate (7.6 mL, 0.16 mol) was added to a solution of (*S*)-2-amino-3-(1*H*-indol-3-yl)propanehydrazide hydrochloride **19** (5.0 g, 19.6 mM) in methanol (100 mL). The reaction mixture was stirred at reflux for 12 h. The solvent was evaporated under reduced pressure, and DCM/^i^PrOH (3:1, 50 mL) was added to the residue. The resulting suspension was triturated by placing the mixture in an ultrasound bath for five minutes, filtered and again placed in the ultrasound bath for another 5 min and filtered. The solvent was removed to yield the product as light brown, sticky solid **10** (98%). The spectral data correspond to those reported in the literature [7].

### 3.3. General Procedure (GP) for Acylhydrazone Formation

GP: To (*S*)-2-amino-3-(1*H*-indol-3-yl)propanehydrazide (**10**) (1 equivalent) was added a small amount of methanol, the corresponding aldehyde (1.2 equivalent), and the resulting mixture was heated at reflux. Upon heating, the starting materials were completely dissolved, subsequently the mixture was heated to reflux overnight. After letting the reaction mixture cool to room temperature, the solvent was evaporated *in vacuo*. The crude product was purified by column chromatography (SiO_2_; MeOH/DCM 0:10 to 1:9), and the corresponding acylhydrazone was obtained in 30%–50% yield [7].

### 3.4. Fluorescence-Based Inhibition Assay

Endothiapepsin was purified from Suparen^®^ (kindly provided by DSM Food Specialties, Heerlen, The Netherlands) by exchanging the buffer to sodium acetate buffer (0.1 M, pH 4.6) using a Vivaspin 500 (Sartorius, Rotterdam, The Netherlands) with a molecular weight cutoff at 10,000 Da. The measurement of the absorption at 280 nm, assuming an extinction coefficient of 1.15 for 1 mg/mL solutions, afforded the protein concentration [19].

Stock solutions (100 mM in DMSO) were prepared for acylhydrazones **2**–**9** (mixture of *E*/*Z* isomers). The final reaction volume was 200 μL containing 0.4 nM endothiapepsin, 1.8 μM substrate and 2.1% DMSO. The final concentration of inhibitors was 1000 μM in the first well and subsequent half dilution in next 10 wells. In the same way, blanks were prepared using DMSO instead of the inhibitor stock solutions. As substrate, Abz–Thr–Ile–Nle-*p*-nitro-Phe–Gln–Arg–NH_2_ (purchased from Bachem, Bubendorf, Switzerland) was used for the fluorescence screening assay. The assay was performed with flat bottom 96-well microplates (purchased from Greiner Bio-One, Alphen aan den Rijn, The Netherlands) using a Synergy Mx microplate reader at an excitation wavelength of 337 nm and an emission wavelength of 414 nm. The *K*_m_ of the substrate toward endothiapepsin was known, 1.6 μM. The assay buffer (0.1 M sodium acetate buffer, pH 4.6, containing 0.001% Tween 20) was premixed with the substrate and inhibitor; endothiapepsin was added directly before the measurement. As the substrate is a fluorogenic substrate, during measurement the fluorescence increased because of substrate hydrolysis by endothiapepsin. The initial slopes of the fluorescence in the acylhydrazone-containing wells were compared to the initial slope of the blanks for data analysis.

### 3.5. Modeling and Docking

One X-ray crystal structure of complexe of endothiapepsin (PDB codes: 4KUP) was used for our modeling [7]. Several acylhydrazones were designed. The energy of the system was minimized using the MAB force field as implemented in the computer program MOLOC, whilst keeping the protein coordinates fixed for the PDB code: 4KUP. In all cases, the acylhydrazone addresses the catalytic dyad directly via hydrogen-bonding interaction. Taking inspiration from the co-crystal structures of endothiapepsin with eleven fragments we have reported previously [20], as well as from hot-spot analysis of the active site of endothiapepsin, several acylhydrazones with different aromatic substituents were designed and subsequent energy minimization (MAB force field, a generally applicable molecular force field for structure modelling in medicinal chemistry) was done using MOLOC to optimize amide–π interactions. All types of interactions (hydrogen bonds, amide–π interactions and lipophilic interactions) between designed acylhydrazones and protein were measured in MOLOC. All the designed acylhydrazones were subsequently docked into the active site of endothiapepsin by using the FlexX docking module in the LeadIT suite. During the docking, the binding site in the protein was restricted to 9 Å around the co-crystallized ligand and the 30 top (FlexX)-scored solutions were retained, and subsequently post-scored with the HYDE module in LeadIT v.2.1.3. After careful visualization to exclude poses with significant inter- or intramolecular clash terms or unfavorable conformations, the resulting solutions were subsequently ranked according to their binding energies. The top-ranked solutions identified in this way were chosen.

## 4. Conclusions

By using SBDD, in particular optimizing an amide–π interaction, we designed a library of eight acylhydrazone-based inhibitors starting from hit **1**. These compounds inhibit the aspartic protease endothiapepsin with IC_50_ values in the low micromolar region. The best compound **2** displays an IC_50_ value of 7.0 µM, which is two-fold more potent than the original hit **1**. The increase in potency could be due to the strengthened amide–π interaction compared to the original hit **1**, owing to the more strongly electron-withdrawing nature of the trifluoromethyl group as well as better lipophilic interactions. Furthermore, **2** also has the potential to have increased metabolic stability than the original hit **1** because of the presence of a trifluoromethyl instead of three methyl groups. The observed structure–activity relationships provide evidence that the inhibitors indeed adopt the predicted binding mode. We will validate this by co-crystallization studies, which will provide useful insights for future structure-based optimization of inhibitors for the real drug targets and insights into molecular recognition.

## Supplementary Materials

Supplementary materials can be found at http://www.mdpi.com/1422-0067/16/08/19184/s1.
