# Supplementary material for: Structure-Based Optimization of Inhibitors of the Aspartic Protease Endothiapepsin
Source: Int J Mol Sci. 2015 Aug 14;16(8):19184–94. doi: 10.3390/ijms160819184 (PMC4581293; doi:10.3390/ijms160819184)
Supplement: Supplementary file 1 [file ijms-16-19184-s001.pdf]

Supplementary Information

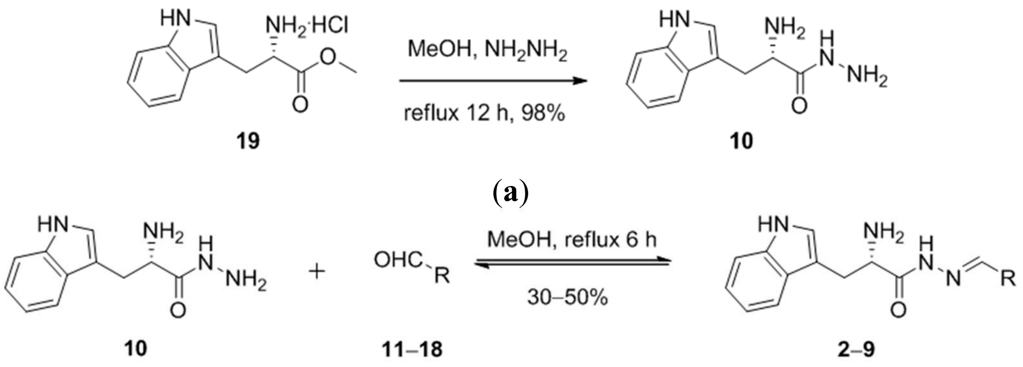

(**a**)

(**b**)

Scheme S1. Synthesis of (**a**) hydrazide **10**; and (**b**) achylhydrazones **2**–**9**.

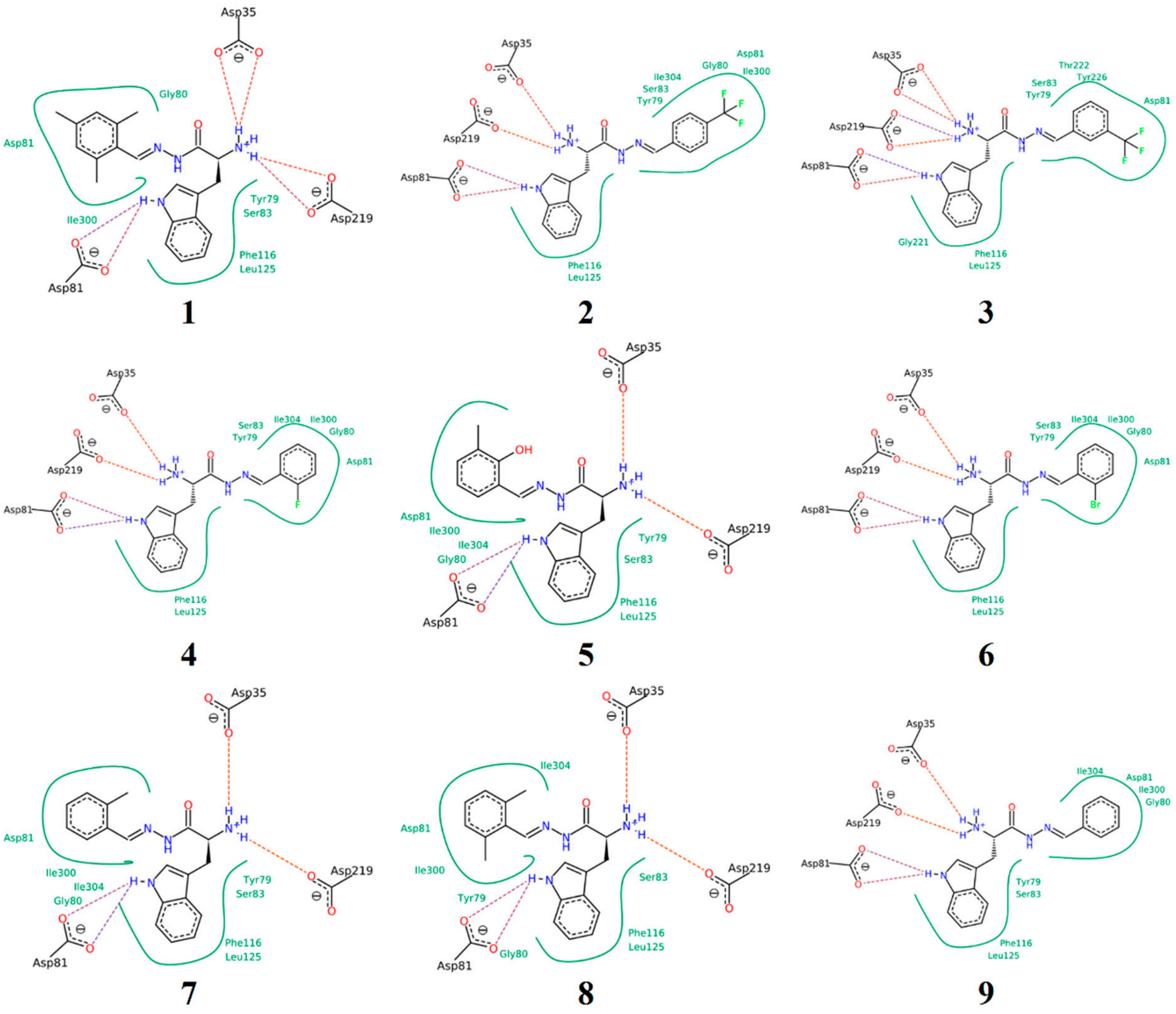

Figure S1. Schematic representation of the predicted binding modes of acylhydrazone-based inhibitors **1**–**9** in the active site of the endothiapepsin. These binding modes are the result of a docking run using the FlexX docking module with 30 poses and represent the top-scoring pose after HYDE scoring and careful visual inspection to exclude poses with significant inter- or intra-molecular clash terms or unfavorable conformations. The figure was generated with PoseView [21] as implemented in the LeadIT suite.

1. Experimental Procedures

*1.1. (S,E)-2-Amino-3-(1H-indol-3-yl)-N'-(4-(trifluoromethyl)benzylidene)propanehydrazide (**2**)*

The acylhydrazone **2** was synthesized according to **GP** by using (*S*)-2-amino-3-(1*H*-indol-3-yl) propanehydrazide (**10**) (408 mg, 1.87 mM) and 4-trifluoromethyl-benzaldehyde **11** (306 µL, 2.24 mM). After purification, the acylhydrazone **2** was obtained as a mixture of *E* and *Z* isomers (*E*:*Z* = 64:36) as a white solid (365 mg, 52%). m.p. 187–190 °C; $[\alpha]_D^{20}$ = +53.7 (*c* = 0.114 in MeOH); ^{1}H NMR (400 MHz, CD_3OD) δ = 8.03 (s, 1H, *E*), 7.92 (d, *J* = 8.2 Hz, 1H), 7.86 (s, 1H, *Z*), 7.70 (d, *J* = 8.2 Hz, 1H), 7.67–7.63 (m, 2H), 7.62 (s, 1H), 7.34 (d, *J* = 8.1 Hz, 1H, *E*), 7.24 (d, *J* = 8.1 Hz, 1H, *Z*), 7.15–7.06 (m, 2H), 7.05–6.97 (m, 1H), 4.74 (t, *J* = 6.7 Hz, 1H, *Z*), 3.73 (t, *J* = 6.7 Hz, 1H, *E*), 3.29–3.22 (m, 1H), 3.17–3.07 (m, 1H); ^{13}C NMR (101 MHz, CD_3OD) δ = 178.5, 174.5, 148.2, 144.1, 139.3, 138.2, 136.3, 129.2, 128.8, 124.7 (d, *J* = 25.9 Hz), 124.86, 124.61, 122.50 (d, *J* = 5.5 Hz), 119.8 (d, *J* = 17.7 Hz), 119.5 (d, *J* = 9.2 Hz), 112.4, 111.3, 111.0, 56.4, 52.7, 32.5 (d, *J* = 13.5 Hz); IR (cm^{-1}): 3283 (br), 3058, 2920, 1671, 1455, 743; ^{19}F NMR (376 MHz, CD_3OD) δ = −64.31, −64.39; HRMS (ESI) calculated for $C_{19}H_{17}F_3N_4O$ [M + H]$^+$: 375.1427, found: 375.1431; Elemental analysis, calculated for $C_{19}H_{17}F_3N_4O$ (%): C 60.96, H 4.58, N 14.97. found: C 60.45, H 4.54, N 14.64.

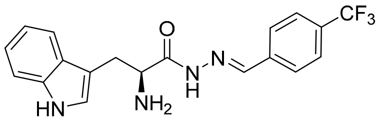

Scheme S2. Structure of (*S*,*E*)-2-amino-3-(1*H*-indol-3-yl)-*N'*-(4-(trifluoromethyl)benzylidene) propanehydrazide (**2**).

*1.2. (S,E)-2-Amino-3-(1H-indol-3-yl)-N'-(3-(trifluoromethyl)benzylidene)propanehydrazide (**3**)*

The acylhydrazone **3** was synthesized according to **GP** by using (*S*)-2-amino-3-(1*H*-indol-3-yl) propanehydrazide (**10**) (403 mg, 1.85 mM) and 3-trifluoromethyl-benzaldehyde **12** (297 µL, 2.22 mM). After purification, the acylhydrazone **3** was obtained as a mixture of *E* and *Z* isomers (*E*:*Z* = 60:40) as a white solid (332 mg, 48%). m.p. 67–71 °C; $[\alpha]_D^{20}$ = +39.1 (*c* = 0.097 in MeOH); ^{1}H NMR (400 MHz, CD_3OD): δ = 8.08 (s, 1H, *E*), 8.00 (s, 1H, *E*), 7.91 (s, 1H, *Z*), 7.89 (d, *J* = 4.1 Hz, 1H, *E*), 7.88 (s, 1H, *Z*), 7.71 (d, *J* = 7.7 Hz, 1H, *Z*), 7.68–7.47 (m, 3H), 7.31 (d, *J* = 8.1 Hz, 1H, *E*), 7.25 (d, *J* = 8.1 Hz, 1H, *Z*), 7.12 (s, 1H, *Z*), 7.11 (s, 1H, *E*), 7.05 (dd, *J* = 15.1, 8.0 Hz, 1H), 7.01–6.95 (m, 1H), 3.76 (t, *J* = 6.8 Hz, 1H, *E*), 3.38–3.22 (m, 1H), 3.16–3.06 (m, 1H); ^{13}C NMR (101 MHz, CD_3OD): δ = 176.0, 173.8, 148.4, 144.9, 138.2 (d, *J* = 8.5 Hz), 136.5 (d, *J* = 15.1 Hz), 133.8, 132.3, 130.7, 130.7 (d, *J* = 210.9 Hz), 130.7, 128.7, 128.6, 127.7 (dd, *J* = 9.5, 5.8 Hz), 127.5 (dd, *J* = 7.8, 4.0 Hz), 125.1 (dd, *J* = 7.8, 3.8 Hz), 124.4, 122.6, 122.5, 120.0, 119.9, 119.4, 119.1, 112.4, 112.3, 110.7, 110.2, 56.2, 52.5, 32.1, 31.1, 25.3; ^{19}F NMR (376 MHz, $CDCl_3$): δ = −62.82; IR (cm^{-1}): 3332 (br), 2497 (br), 1668, 1326, 1120; HRMS (ESI) calculated for $C_{19}H_{17}F_3N_4O$ [M + H]$^+$: 375.1427, found: 375.1429.

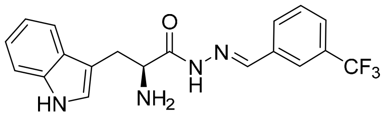

Scheme S3. Structure of (*S*,*E*)-2-amino-3-(1*H*-indol-3-yl)-*N*′-(3-(trifluoromethyl)benzylidene) propanehydrazide (**3**).

*1.3. (S,E)-2-Amino-N'-(2-fluorobenzylidene)-3-(1H-indol-3-yl)propanehydrazide (**4**)*

The acylhydrazone **4** was synthesized according to **GP** by using (*S*)-2-amino-3-(1*H*-indol-3-yl) propanehydrazide (**10**) (363 mg, 1.66 mM) and 2-fluorobenzaldehyde **13** (210 μL, 1.99 mM). After purification, the acylhydrazone **4** was obtained as a mixture of *E* and *Z* isomers (*E*:*Z* = 58:42) as a white solid (237 mg, 44%). m.p. 175–176 °C; $[\alpha]_D^{20}$ = +75.5 (*c* = 0.200 in MeOH); ^{1}H NMR (400 MHz, CD_3OD) δ = 8.27 (s, 1H, *E*), 8.17–8.08 (m, 1H), 7.71 (d, *J* = 7.5 Hz, 1H), 7.68 (d, *J* = 3.4 Hz, 1H), 7.67–7.60 (m, 1H), 7.49–7.39 (m, 1H), 7.34 (d, *J* = 8.0 Hz, 1H), 7.31–6.94 (m, 9H), 4.73 (t, *J* = 6.6 Hz, 1H, *Z*), 3.72 (t, *J* = 6.6 Hz, 1H, *E*), 3.26 (m, 1H), 3.17–3.00 (m, 1H); ^{13}C NMR (101 MHz, CD_3OD) δ = 178.1, 174.2, 164.2, 161.7, 142.7, 139.1, 139.0, 138.2, 133.4 (d, *J* = 8.6 Hz), 132.8 (d, *J* = 8.5 Hz), 128.8 (d, *J* = 9.5 Hz), 128.7, 128.1, 125.7, 124.7 (d, *J* = 16.2 Hz), 123.0, 122.5, 119.8 (d, *J* = 15.3 Hz), 119.5, 116.7 (dd, *J* = 21.2, 8.6 Hz), 112.4, 111.2, 110.9, 56.3, 52.6, 32.3; ^{19}F NMR (376 MHz, CD_3OD) δ = −123.17 (m), −123.34 (m); IR (cm^{-1}): 3286 (br), 3056, 2921, 1673, 1615, 1455, 1357, 1238, 743; HRMS (ESI) calculated for $C_{18}H_{17}FN_4O$ $[M + H]^+$: 325.1459, found: 325.1465.

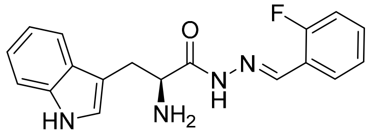

Scheme S4. Structure of (*S*,*E*)-2-amino-*N*′-(2-fluorobenzylidene)-3-(1*H*-indol-3-yl)propanehydrazide (**4**).

*1.4. (S,Z)-2-Amino-N'-(2-hydroxy-3-methylbenzylidene)-3-(1H-indol-3-yl)propanehydrazide (**5**)*

The acylhydrazone **5** was synthesized according to **GP** by using (*S*)-2-amino-3-(1*H*-indol-3-yl) propanehydrazide (**10**) (410 mg, 1.88 mM) and 2-hydroxy-3-methylbenzaldehyde **14** (273 μL, 2.25 mM). After purification, the acylhydrazone **5** was obtained as a mixture of *E* and *Z* isomers (*E*:*Z* = 93:7) as a yellow solid (246 mg, 39%). m.p. 86–90 °C; $[\alpha]_D^{20}$ = +103.0 (*c* = 0.146 in MeOH); ^{1}H NMR (400 MHz, CD_3OD) δ = 8.28 (s, 1H, *E*), 8.11 (s, 1H, *Z*), 7.97 (s, 1H, *E*), 7.63 (d, *J* = 8.0 Hz, 1H), 7.34 (d, *J* = 8.0 Hz, 1H), 7.19 (d, *J* = 7.3 Hz, 1H), 7.13 (s, 1H), 7.11–7.05 (m, 2H), 7.04–6.98 (m, 1H), 6.81 (dd, *J* = 10.0, 5.1 Hz, 1H), 3.72 (t, *J* = 6.7 Hz, 1H, *Z*), 3.27 (dd, *J* = 14.2, 6.8 Hz, 1H), 3.12 (dd, *J* = 14.2, 6.8 Hz, 1H), 2.26 (s, 3H); ^{13}C NMR (101 MHz, CD_3OD) δ = 173.3, 157.6, 152.6, 149.6, 138.1, 134.0, 130.0, 128.7, 126.9, 124.8, 122.5, 120.1, 119.9, 119.4, 118.2, 112.3, 111.0, 56.3, 32.4, 15.7; IR (cm^{-1}): 3351 (br), 2475 (br), 2216, 2071, 1120, 972; HRMS (ESI) calculated for $C_{19}H_{20}N_4O_2$ $[M + H]^+$: 337.1659, found: 337.1664.

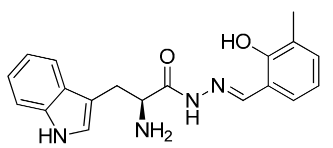

Scheme S5. Structure of (*S*,*Z*)-2-amino-*N*′-(2-hydroxy-3-methylbenzylidene)-3-(1*H*-indol-3-yl) propanehydrazide (**5**).

*1.5. (S,E)-2-Amino-N'-(2-bromobenzylidene)-3-(1H-indol-3-yl)propanehydrazide (**6**)*

The acylhydrazone **6** was synthesized according to **GP** by using (*S*)-2-amino-3-(1H-indol-3-yl) propanehydrazide (**10**) (345 mg, 1.58 mM) and 2-bromobenzaldehyde **15** (220 µL, 1.89 mM). After purification, the acylhydrazone **6** was obtained as a mixture of *E* and *Z* isomers (*E*:*Z* = 55:45) as a white solid (315 mg, 52%). m.p. 80–86 °C; $[\alpha]_D^{20}$ = +54.8 (*c* = 0.091 in MeOH); ^{1}H NMR (400 MHz, CD_3OD) δ = 7.98 (s, 1H, *E*), 7.92 (s, 1H, *E*), 7.83 (s, 1H, *Z*), 7.82 (s, 1H, *Z*), 7.68 (d, *J* = 6.0 Hz, 1H, *E*), 7.66–7.61 (m, 1H), 7.56 (ddd, *J* = 7.9, 2.8, 1.8 Hz, 1H), 7.49 (d, *J* = 7.9 Hz, 1H, *Z*), 7.37–7.31 (m, 1H), 7.31–7.26 (m, 1H), 7.13 (d, *J* = 4.3 Hz, 1H), 7.10–7.05 (m, 1H), 7.01 (t, *J* = 7.4 Hz, 1H), 4.77 (t, *J* = 7.7 Hz, 1H, *Z*), 3.73 (t, *J* = 6.7 Hz, 1H, *E*), 3.31–3.24 (m, 1H), 3.17–3.00 (m, 1H); ^{13}C NMR (101 MHz, CD_3OD) δ = 177.4, 174.1, 148.3, 144.6, 138.2, 138.1, 137.7, 134.2, 134.0, 133.4, 131.6, 131.5, 131.1, 130.3, 128.7, 127.7, 127.4, 124.8, 123.8, 123.5, 122.5, 122.5, 120.1, 119.9, 119.4, 119.3, 112.4, 112.3, 110.9, 110.8, 56.3, 52.5, 32.3, 32.0; IR (cm^{-1}): 3287 (br), 3056, 2920, 1673, 1561, 744; HRMS (ESI) calculated for $C_{18}H_{17}BrN_4O$ [M + H]$^+$: 387.0638, found: 387.0639.

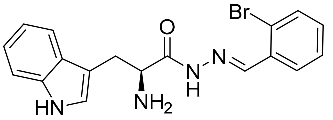

Scheme S6. Structure of (*S*,*E*)-2-amino-*N*′-(2-bromobenzylidene)-3-(1*H*-indol-3-yl)propanehydrazide (**6**).

1.6. (S,E)-2-Amino-3-(1H-indol-3-yl)-N'-(2-methylbenzylidene)propanehydrazide (7)

The acylhydrazone **7** was synthesized according to **GP** by using (*S*)-2-amino-3-(1*H*-indol-3-yl) propanehydrazide (**10**) (200 mg, 0.92 mM) and *o*-tolualdehyde **16** (160 µL, 1.38 mM). After purification, the acylhydrazone **7** was obtained as a mixture of *E* and *Z* isomers (*E*:*Z* = 60:40) as a white solid (130 mg, 44%). m.p. 96–98 °C; $[\alpha]_D^{20}$ = +38.4 (*c* = 0.208 in MeOH); ^{1}H NMR (400 MHz, CD_3OD) δ = 8.29 (s, 1H, *E*), 8.23 (s, 1H, *Z*), 7.95 (d, *J* = 7.5 Hz, 1H, *E*), 7.70 (d, *J* = 7.6 Hz, 1H, *Z*), 7.68–7.59 (m, 1H), 7.37–7.25 (m, 2H), 7.25–7.15 (m, 2H), 7.13 (d, *J* = 5.7 Hz, 1H), 7.11–7.05 (m, 1H), 7.05–6.94 (m, 1H), 4.73 (dd, *J* = 7.7, 5.5 Hz, 1H, *Z*), 3.71 (t, *J* = 6.8 Hz, 1H, *E*), 3.31–3.21 (m, 1H), 3.16–3.01 (m, 1H), 2.44 (s, 1H), 2.38 (s, 2H); ^{13}C NMR (101 MHz, $CDCl_3$) δ = 171.0, 147.0, 137.3, 136.5, 136.4, 131.8, 131.1, 130.8, 130.3, 130.1, 127.6, 127.1, 126.4, 123.7, 123.5, 122.1, 122.1, 119.7, 119.4, 119.1, 118.9, 111.5, 55.3, 52.1, 30.7, 25.4, 19.9, 19.5; IR (cm^{-1}): 3300 (br), 3057, 2923, 2461 (br), 1667, 1455, 744; HRMS (ESI) calculated for $C_{19}H_{20}N_4O$ [M + H]$^+$: 321.1710, found: 321.1714.

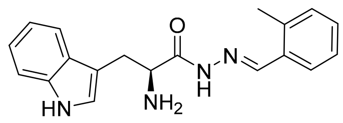

Scheme S7. Structure of (*S*,*E*)-2-amino-3-(1*H*-indol-3-yl)-*N*′-(2-methylbenzylidene)propanehydrazide (**7**).

*1.7. (S,E)-2-Amino-N'-(2,6-dimethylbenzylidene)-3-(1H-indol-3-yl)propanehydrazide (**8**)*

The acylhydrazone **8** was synthesized according to **GP** by using (*S*)-2-amino-3-(1*H*-indol-3-yl) propanehydrazide (**10**) (136 mg, 0.62 mM) and 2,6-dimethylbenzaldehyde **17** (114 mg, 0.85 mM). After purification, the acylhydrazone **8** was obtained as a mixture of *E* and *Z* isomers (*E*:*Z* = 42:50) as a white solid (76 mg, 37%). m.p. 90–97 °C; $[\alpha]_D^{20}$ = +28.6 (*c* = 0.084 in MeOH); ^{1}H NMR (400 MHz, CD_3OD) δ = 8.35 (s, 1H, *E*), 8.30 (s, 1H, *Z*), 7.64 (d, *J* = 8.0 Hz, 1H, *Z*), 7.52 (d, *J* = 8.1 Hz, 1H, *E*), 7.35 (d, *J* = 8.0 Hz, 1H, *Z*), 7.30 (d, *J* = 8.1 Hz, 1H, *E*), 7.23–6.99 (m, 6H), 6.89–6.82 (m, 1H, *E*), 4.65 (dd, *J* = 7.6, 5.3 Hz, 1H, *E*), 3.72 (t, *J* = 6.8 Hz, 1H, *Z*), 3.30–3.21 (m, 1H), 3.18–3.04 (m, 1H), 2.43 (s, 3H), 2.37 (s, 3H); ^{13}C NMR (101 MHz, CD_3OD) *δ* = 177.1, 173.6, 150.2, 146.6, 138.9, 138.8, 138.2, 132.4, 132.2, 130.2, 130.1, 129.8, 129.5, 128.8, 128.8, 124.9, 124.7, 122.5, 122.4, 119.9, 119.7, 119.5, 112.3, 110.9, 110.8, 56.3, 52.9, 32.5, 31.5, 21.5, 21.1; IR (cm^{-1}): 3283 (br), 3058, 2971, 2922, 1672, 1334, 1237, 742; HRMS (ESI) calculated for $C_{20}H_{22}N_4O$ $[M + H]^+$: 335.1866, found: 335.1870.

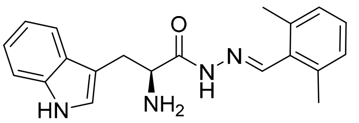

Scheme S8. Structure of (*S*,*E*)-2-amino-*N*′-(2,6-dimethylbenzylidene)-3-(1*H*-indol-3-yl)propanehydrazide (**8**).

*1.8. (S,E)-2-Amino-N'-benzylidene-3-(1H-indol-3-yl)propanehydrazide (**9**)*

The acylhydrazone **9** was synthesized according to **GP** by using (*S*)-2-amino-3-(1*H*-indol-3-yl) propanehydrazide **10** (213 mg, 0.98 mM) and benzaldehyde **18** (120 µL, 1.18 mM). After purification, the acylhydrazone **9** was obtained as a mixture of *E* and *Z* isomers (*E*:*Z* = 52:48) as a white solid (117 mg, 39%). m.p. 146–149 °C; $[\alpha]_D^{20}$ = +62.3 (*c* = 0.132 in MeOH); ^{1}H NMR (400 MHz, CD_3OD) δ = 7.92 (s, 1H, *E*), 7.86 (s, 1H, *Z*), 7.69–7.55 (m, 3H), 7.38–7.26 (m, 4H), 7.11–6.95 (m, 3H), 4.76–4.69 (m, 1H, *Z*), 3.71 (t, *J* = 6.8 Hz, 1H, *E*), 3.29–3.21 (m, 1H), 3.11–2.98 (m, 1H); ^{13}C NMR (101 MHz, CD_3OD) δ = 177.3, 173.8, 150.3, 146.5, 138.0, 135.3, 135.2, 131.5, 131.1, 129.7, 128.7, 128.2, 124.8, 122.5, 119.9, 119.8, 119.4, 56.1, 52.5, 32.2, 31.8; IR (cm^{-1}): 3280 (br), 3058, 2922, 1670, 1455, 743, 692; HRMS (ESI) calculated for $C_{18}H_{18}N_4O$ $[M + H]^+$: 307.1553, found: 307.1557.

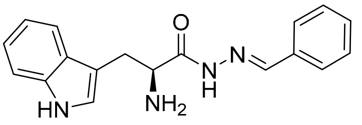

Scheme S9. Structure of (*S*,*E*)-2-amino-*N*′-benzylidene-3-(1*H*-indol-3-yl)propanehydrazide (**9**).

2. NMR Spectra

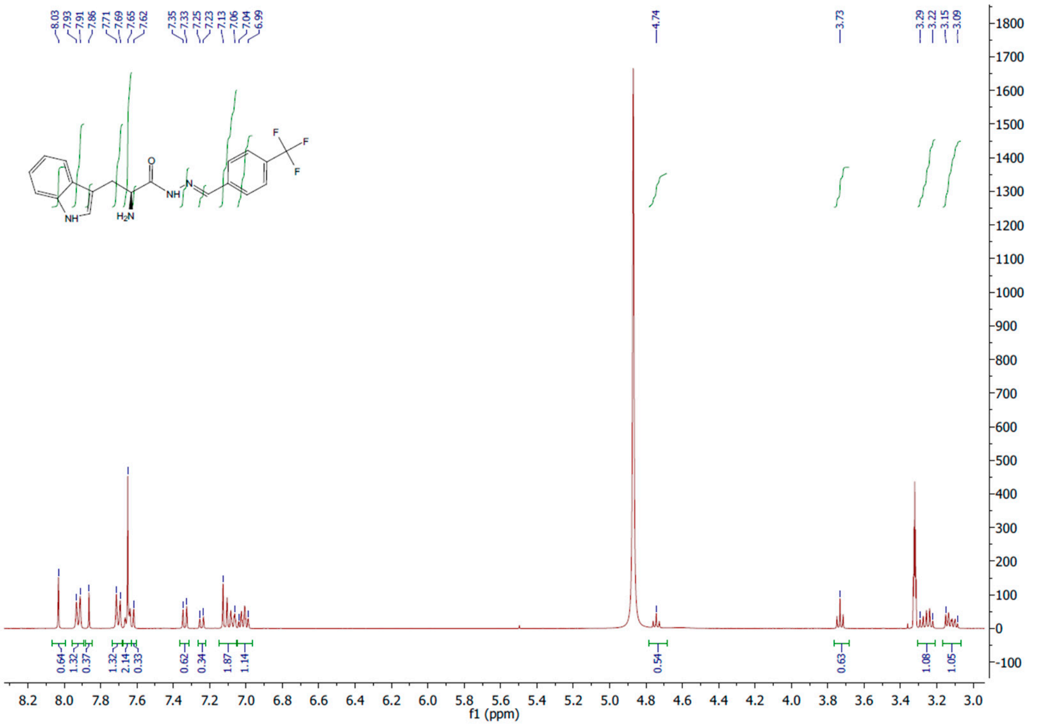

Figure S2. ^{1}H NMR spectrum of compound **2**.

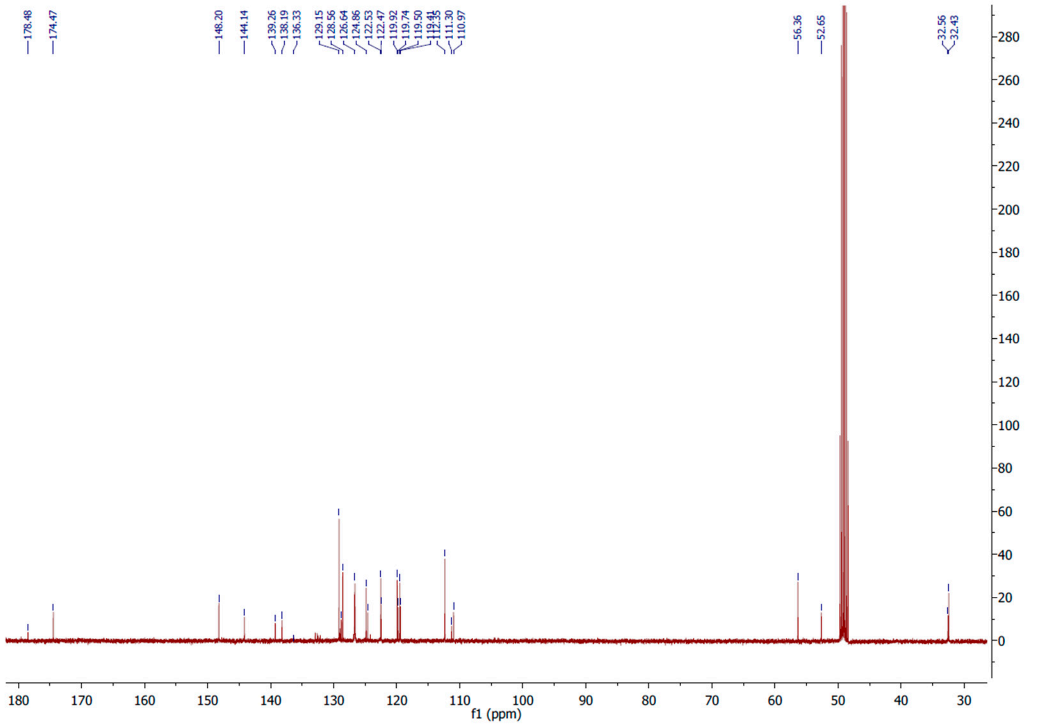

Figure S3. ^{13}C NMR spectrum of compound **2**.

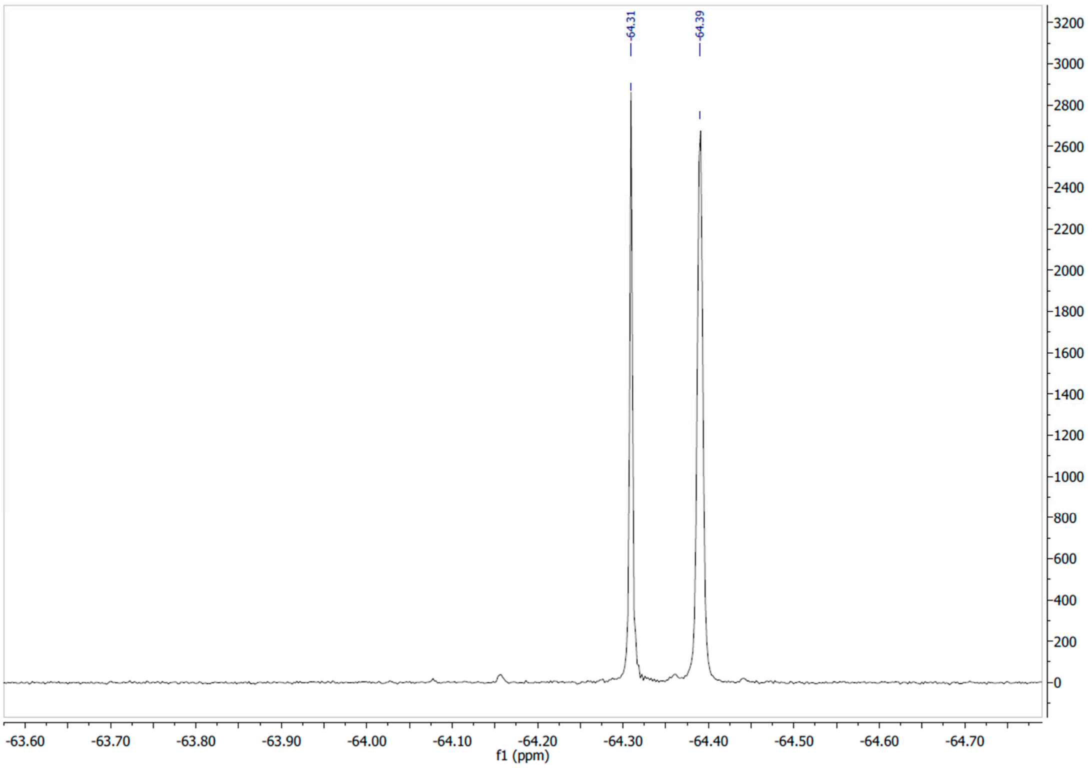

Figure S4. ^{19}F NMR spectrum of compound **2**.

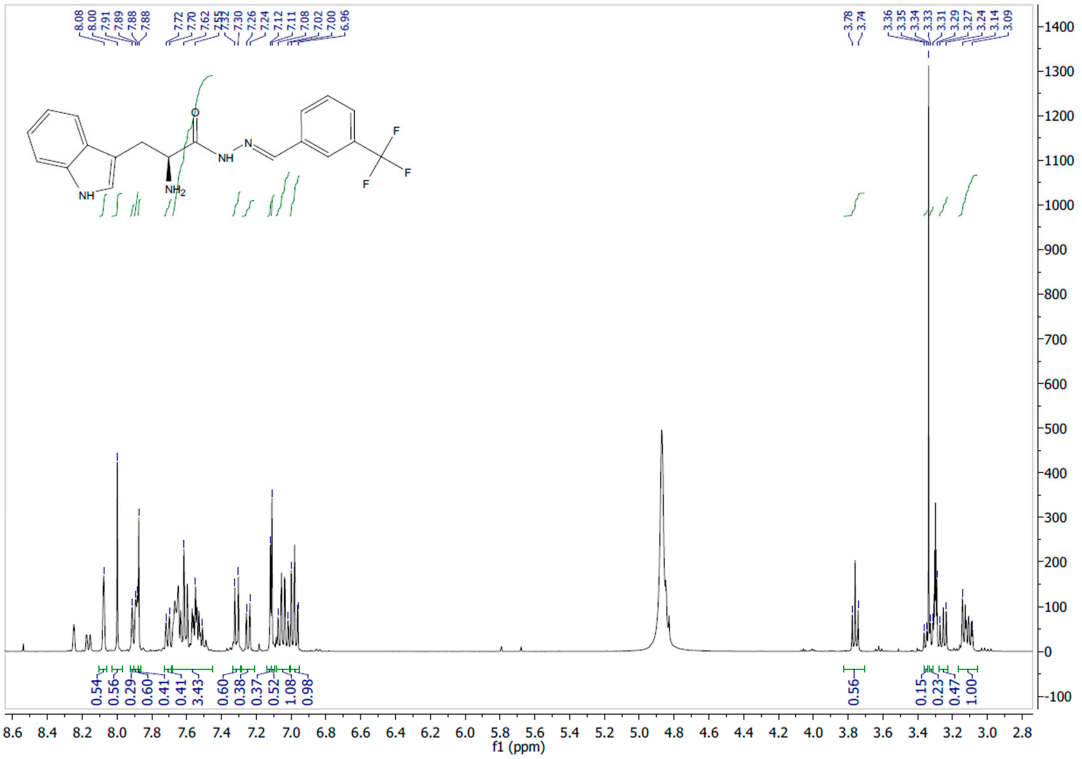

Figure S5. ^{1}H NMR spectrum of compound **3**.

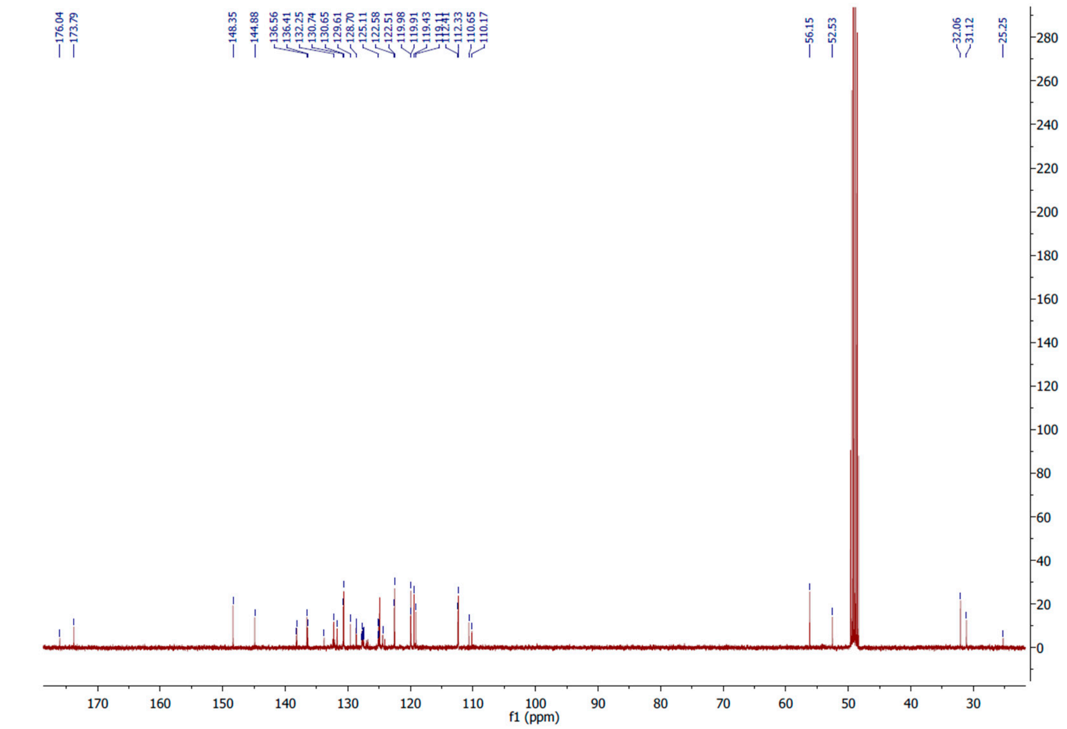

Figure S6. ^{13}C NMR spectrum of compound **3**.

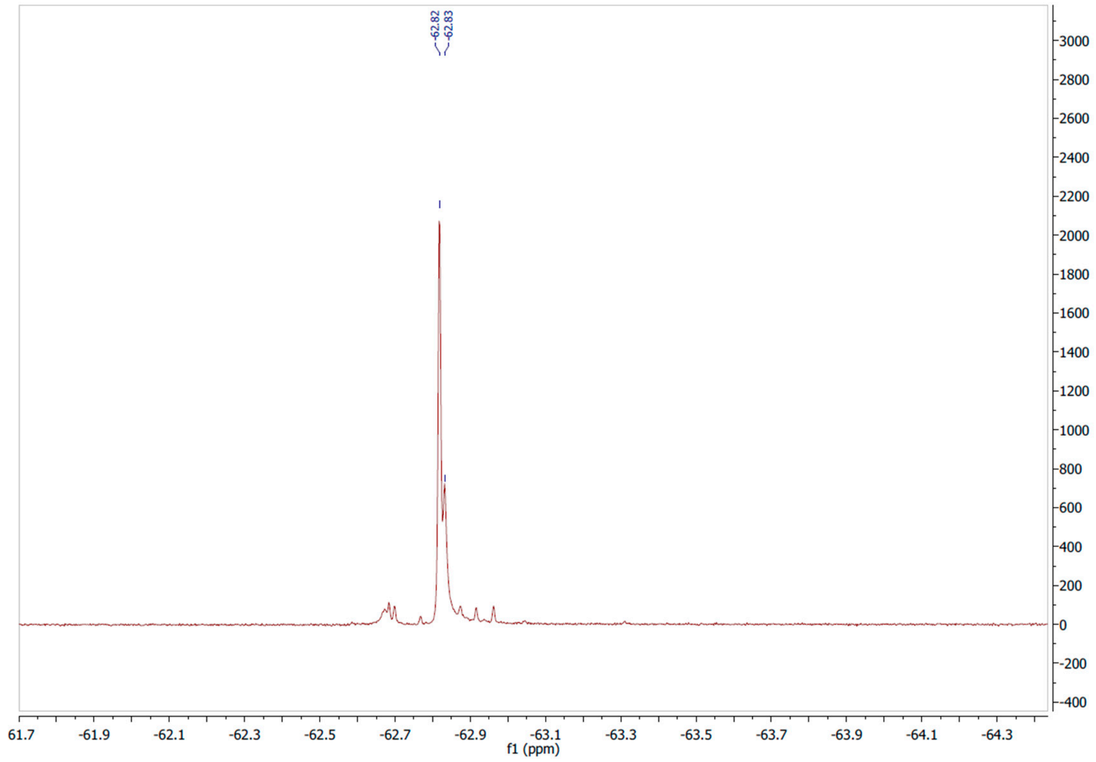

Figure S7. ^{19}F NMR spectrum of compound **3**.

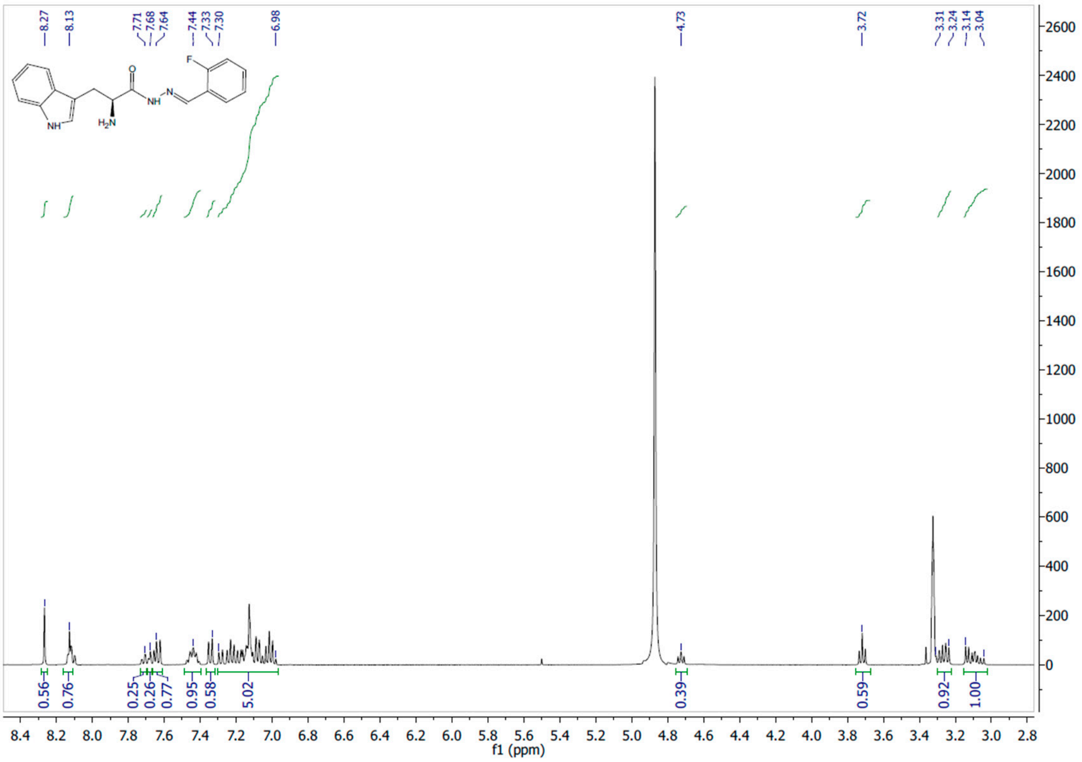

Figure S8. ^{1}H NMR spectrum of compound **4**.

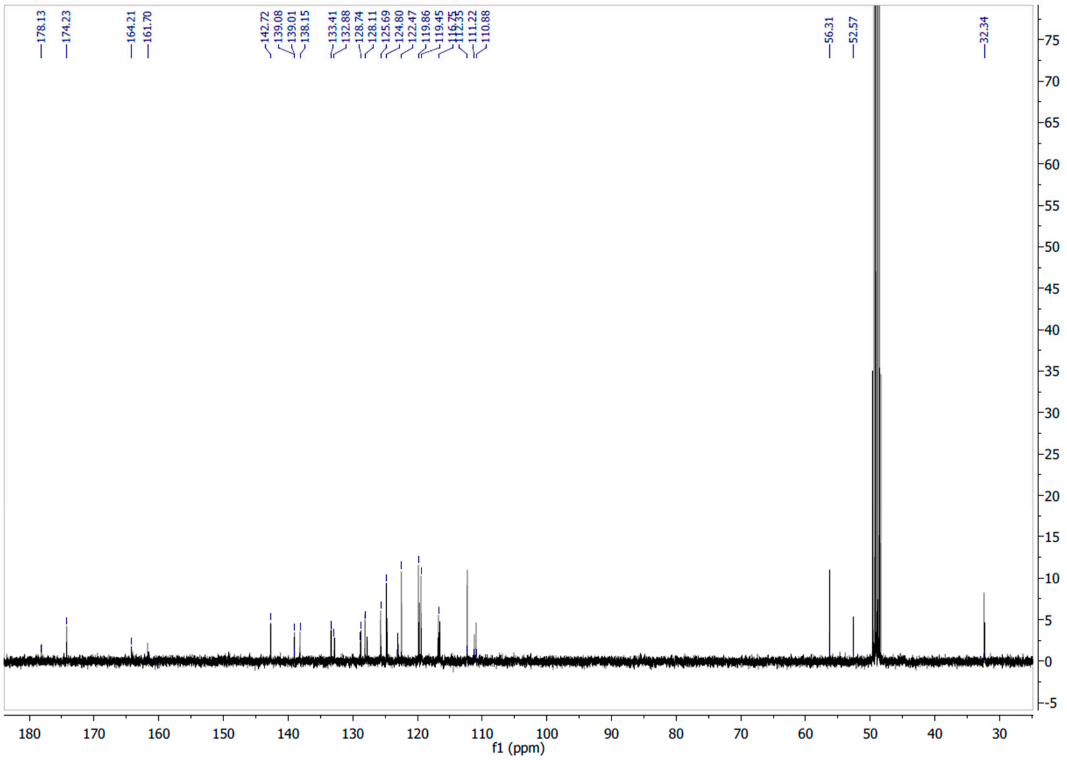

Figure S9. ^{13}C NMR spectrum of compound **4**.

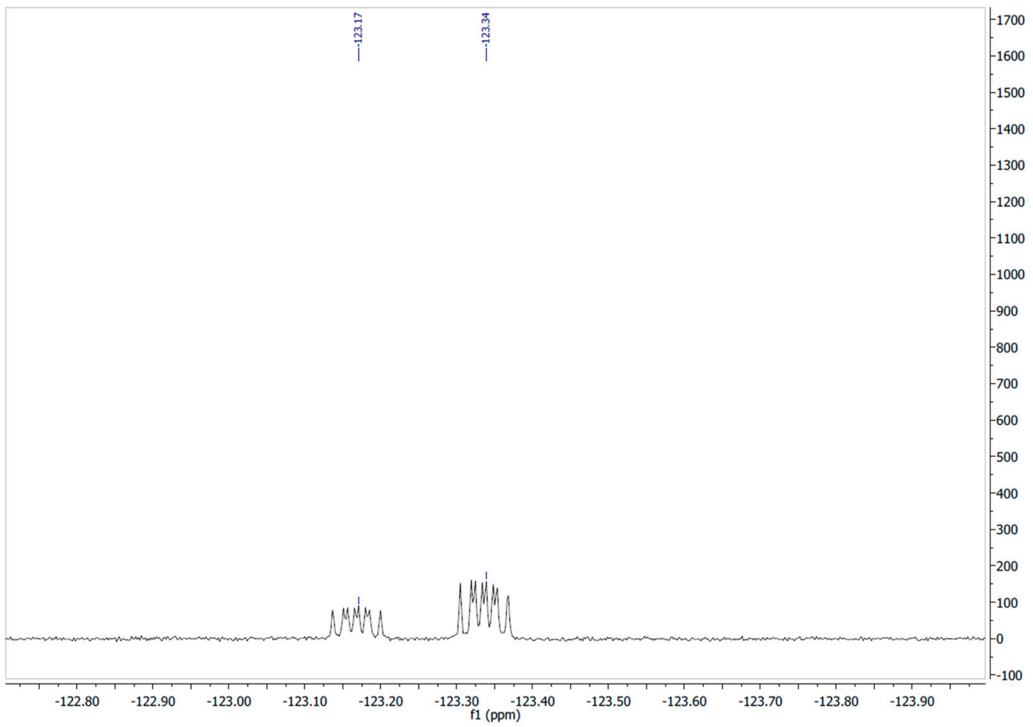

Figure S10. ^{19}F NMR spectrum of compound **4**.

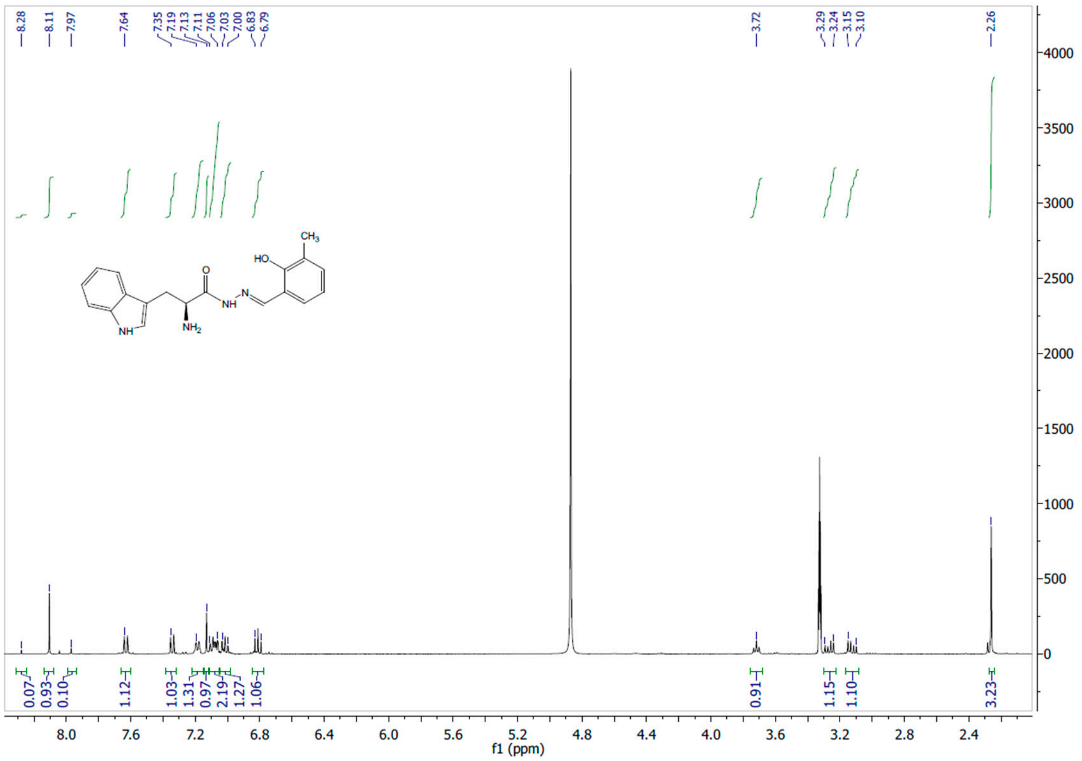

Figure S11. ^{1}H NMR spectrum of compound **5**.

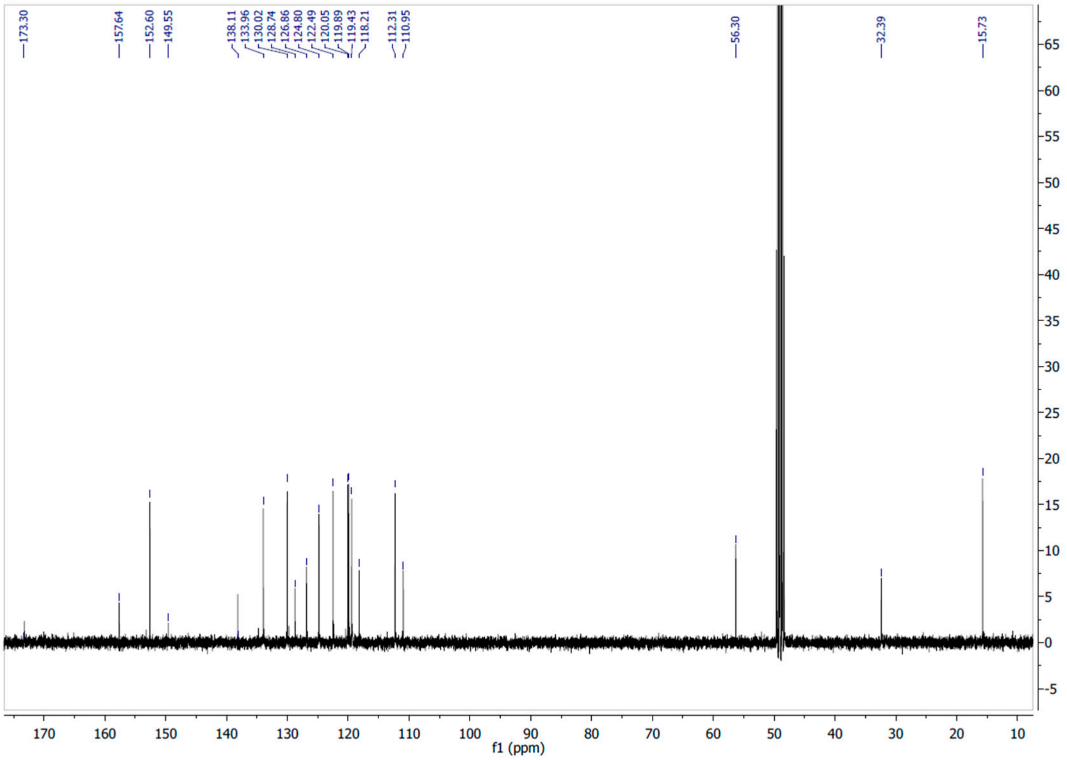

Figure S12. ^{13}C NMR spectrum of compound **5**.

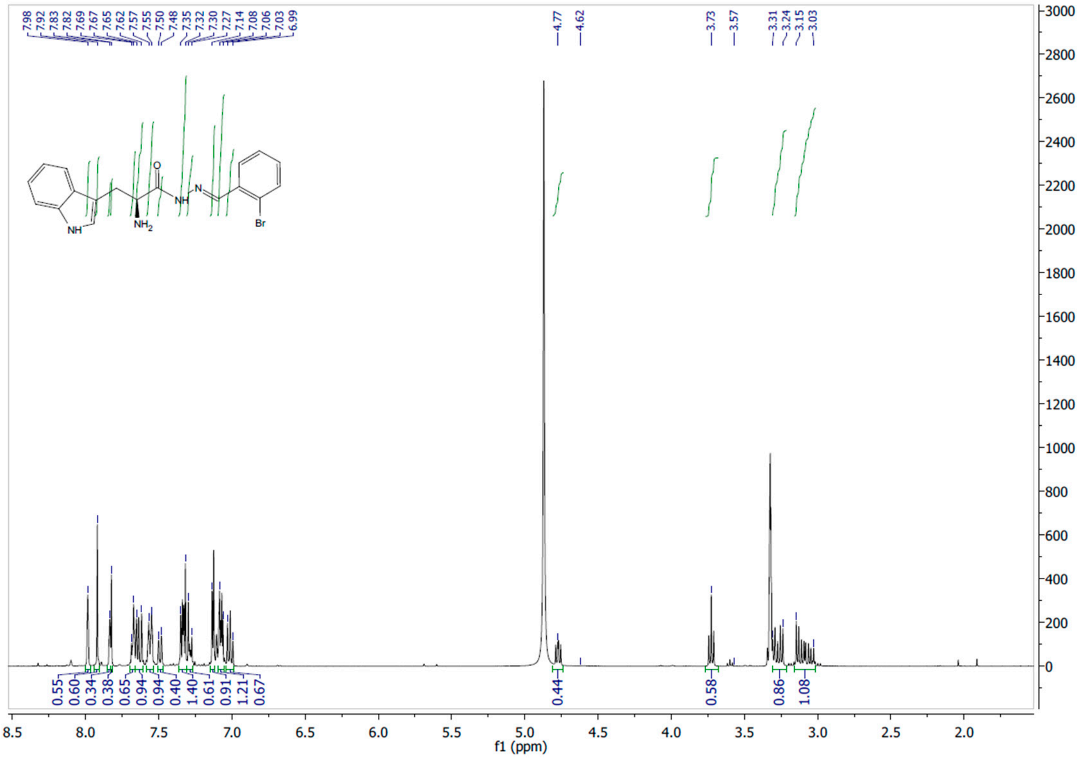

Figure S13. ^{1}H NMR spectrum of compound **6**.

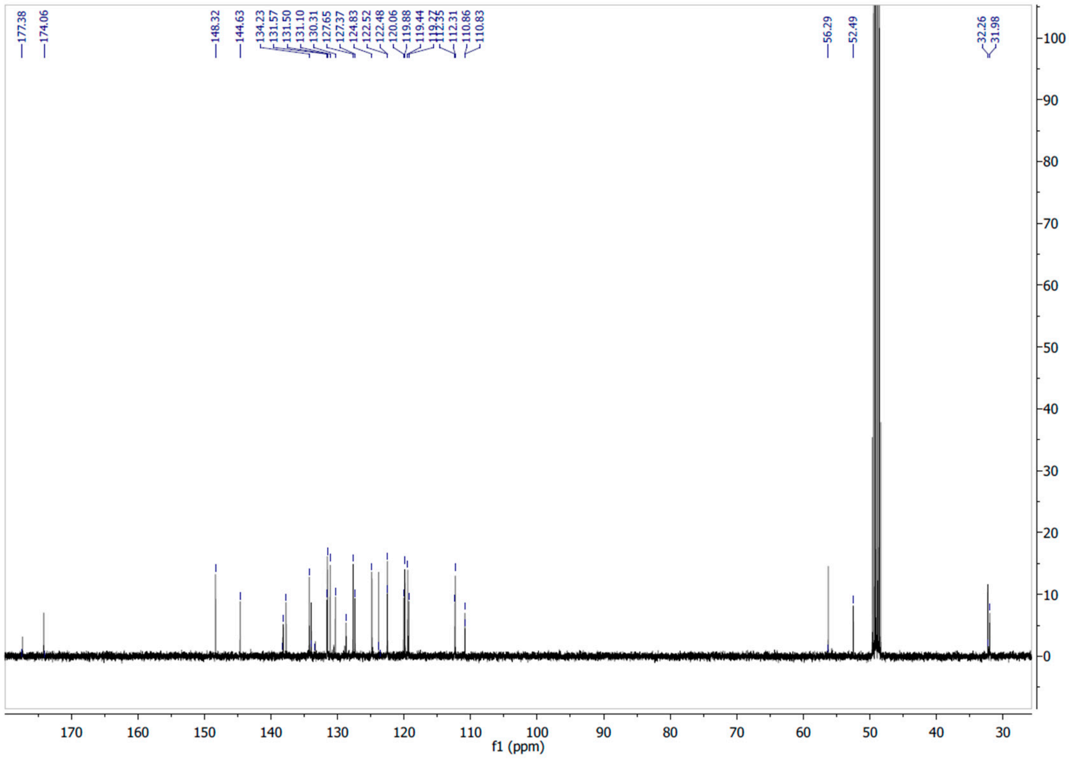

Figure S14. ^{13}C NMR spectrum of compound **6**.

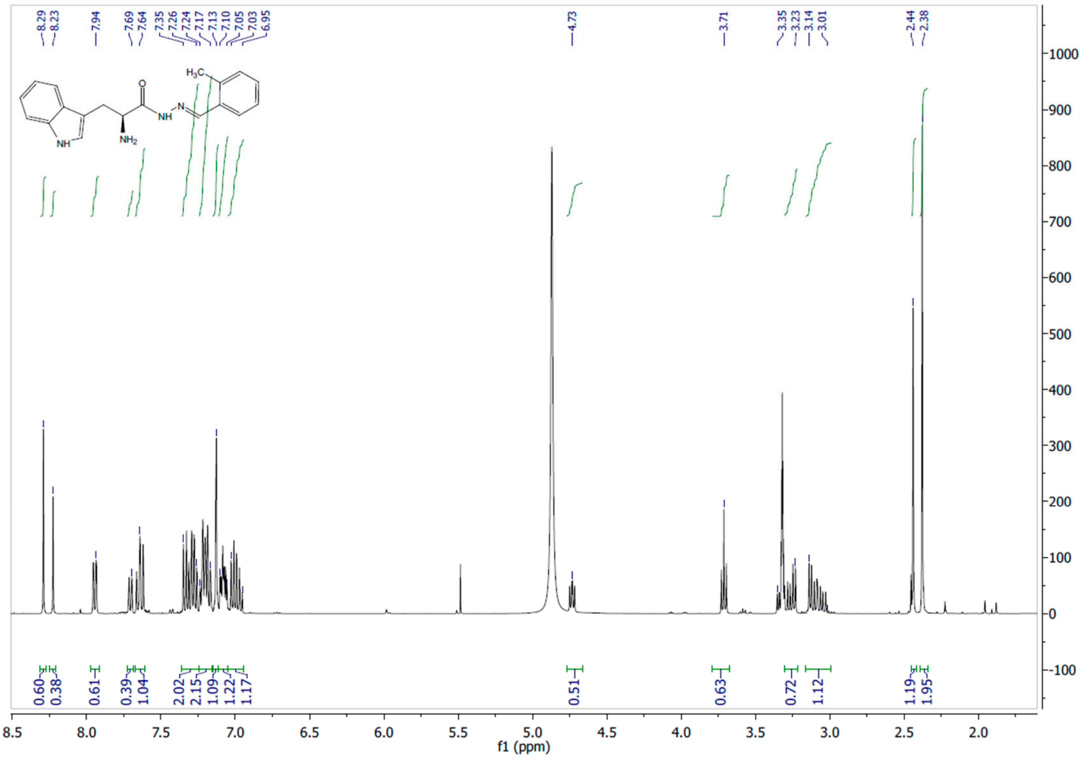

Figure S15. ^{1}H NMR spectrum of compound **7**.

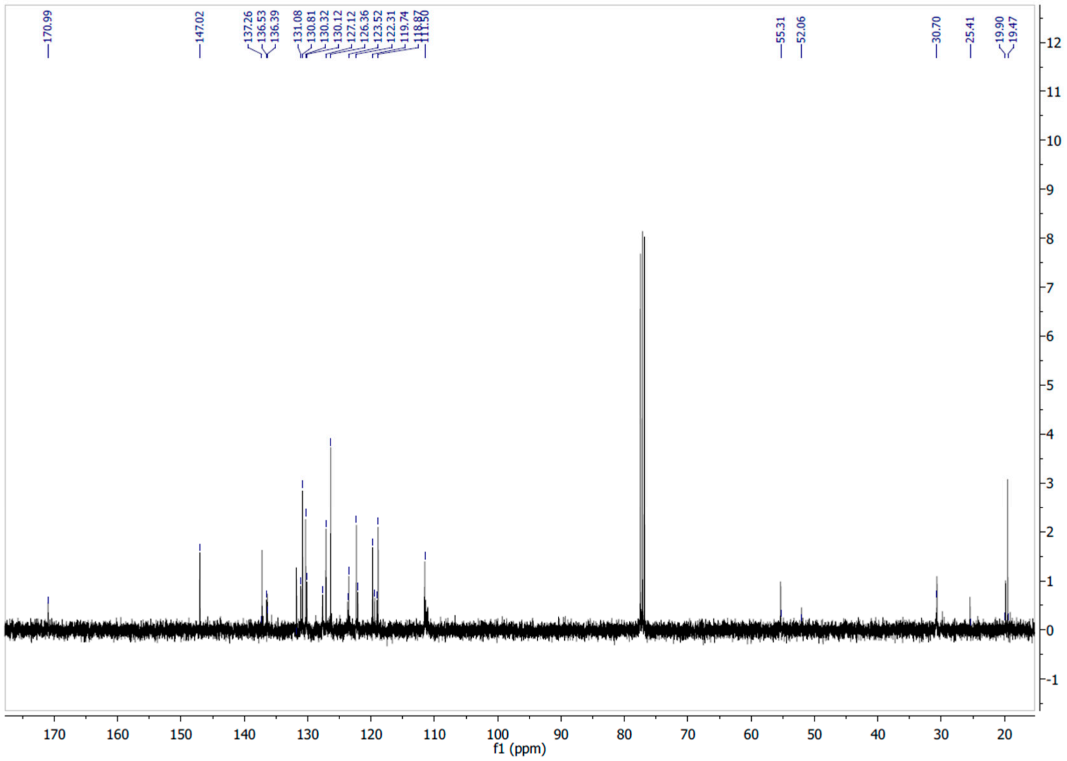

Figure S16. ^{13}C NMR spectrum of compound **7**.

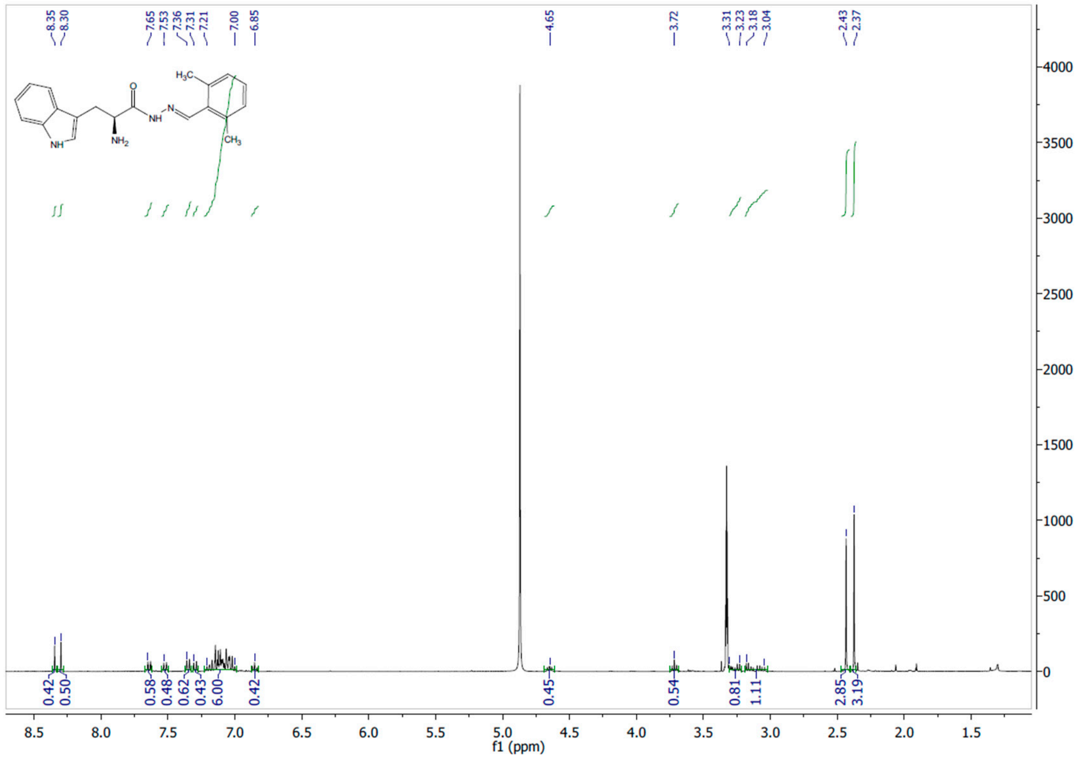

Figure S17. ^{1}H NMR spectrum of compound **8**.

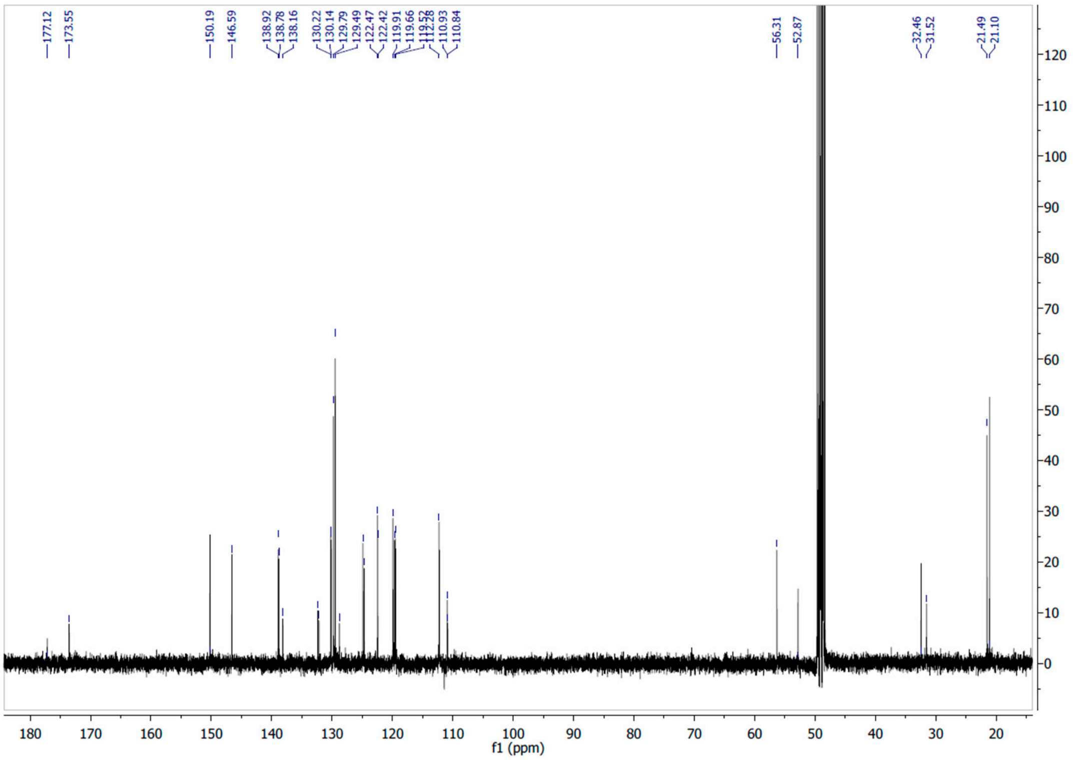

Figure S18. ^{13}C NMR spectrum of compound **8**.

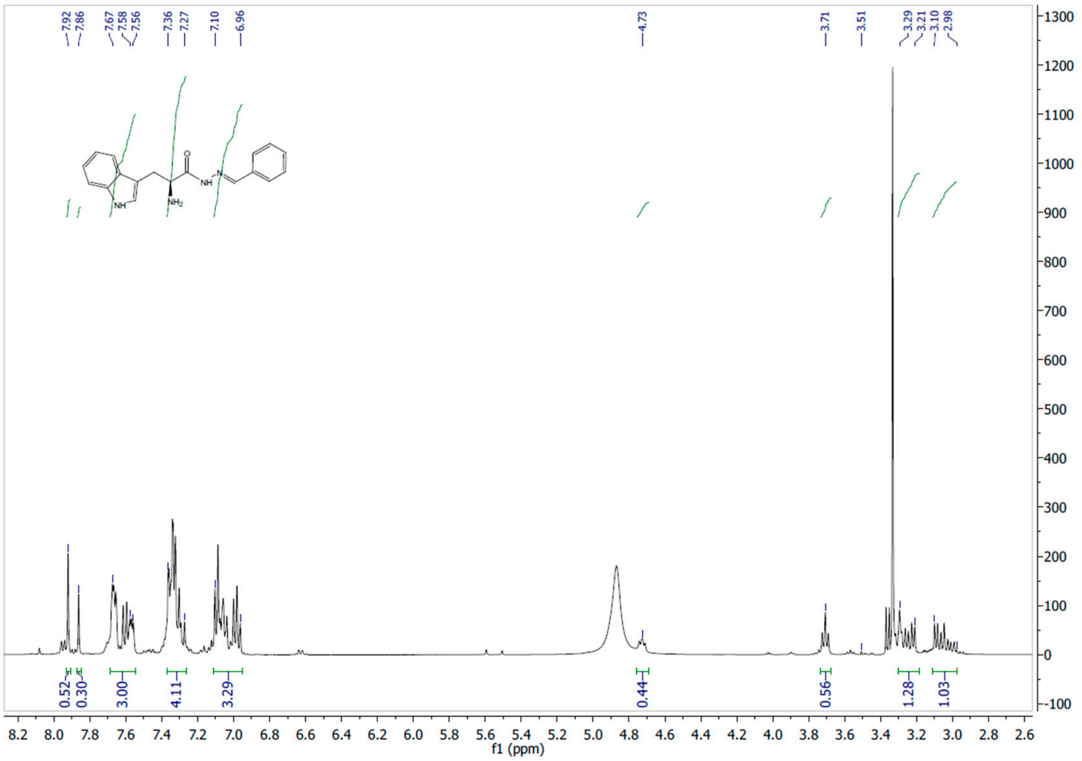

Figure S19. ^{1}H NMR spectrum of compound **9**.

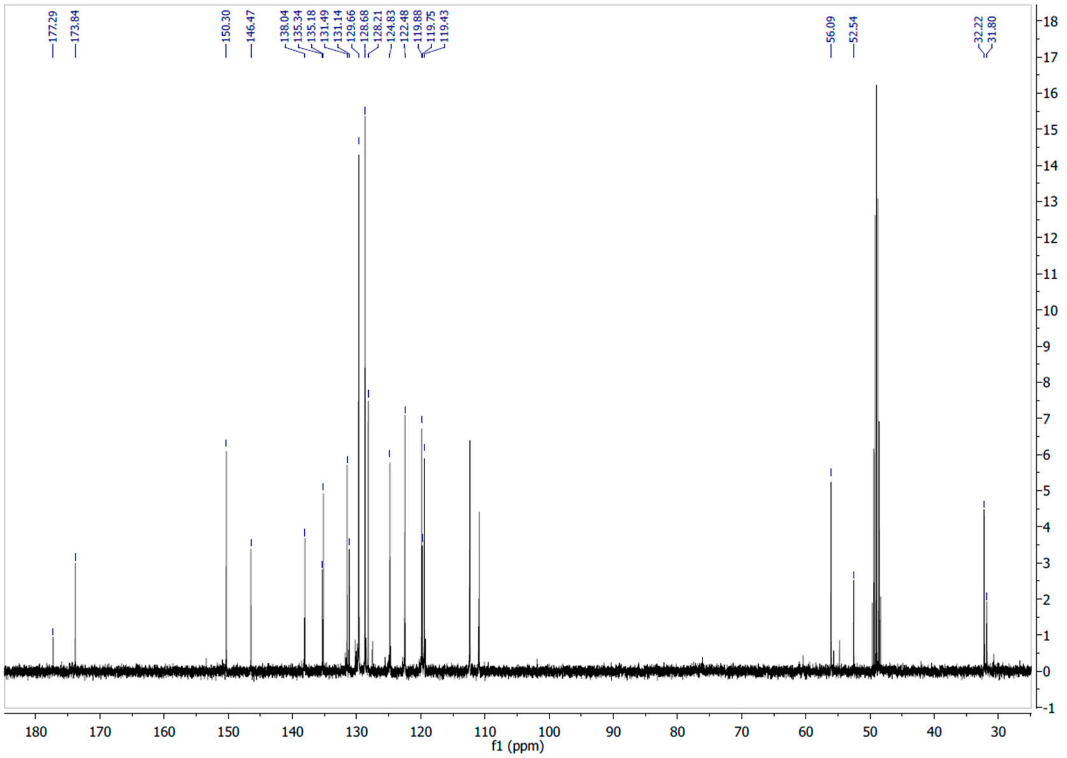

Figure S20. ^{13}C NMR spectrum of compound **9**.

3. HPLC Chromatograms

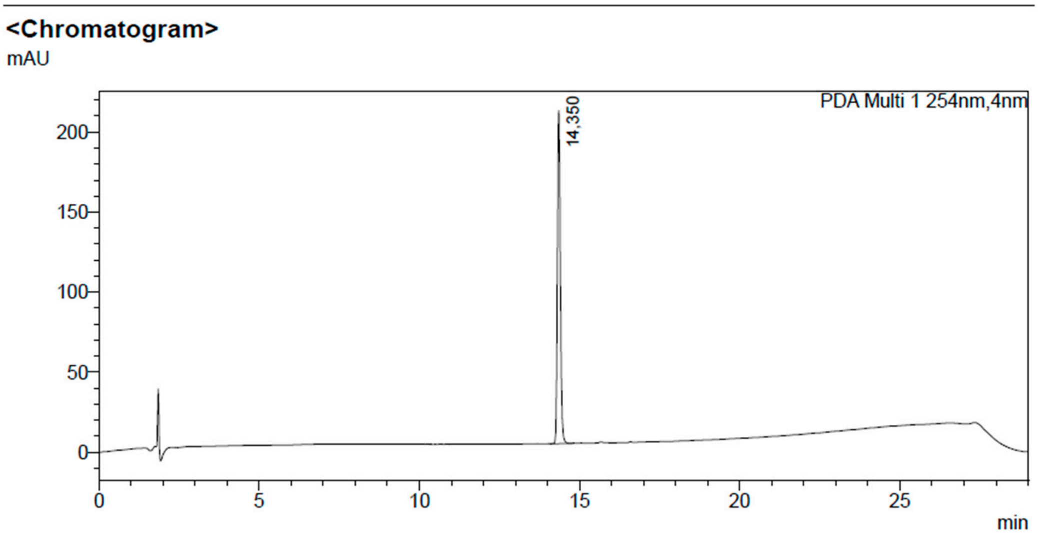

<Peak Table>

PDA Ch1 254nm

Peak#	Ret. Time	Area	Height	Conc.	Unit	Mark	Name
1	14,350	1338261	208193	100,000			
Total		1338261	208193				

Figure S21. Chromatogram of compound **2**.

<Chromatogram>

mAU

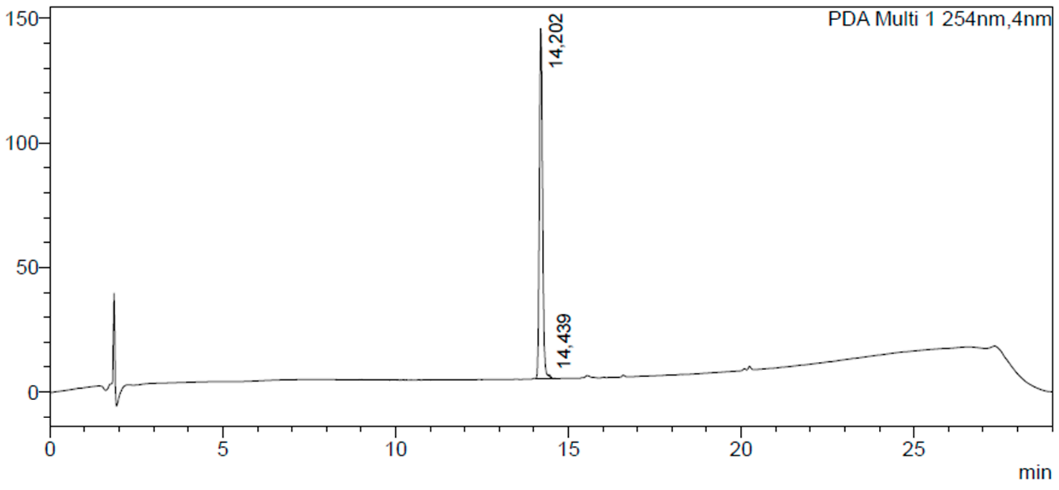

<Peak Table>

PDA Ch1 254nm

Peak#	Ret. Time	Area	Height	Conc.	Unit	Mark	Name
1	14,202	892868	140530	99,766		S	
2	14,439	2094	655	0,234		T	
Total		894963	141185				

Figure S22. Chromatogram of compound **3**.

<Chromatogram>

mAU

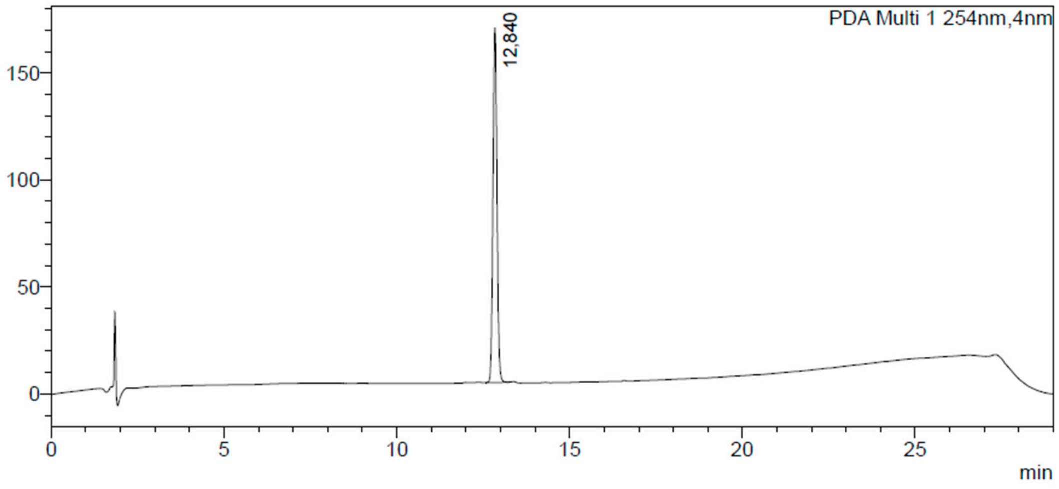

<Peak Table>

PDA Ch1 254nm

Peak#	Ret. Time	Area	Height	Conc.	Unit	Mark	Name
1	12,840	1335566	165876	100,000			
Total		1335566	165876				

Figure S23. Chromatogram of compound **4**.

<Chromatogram>

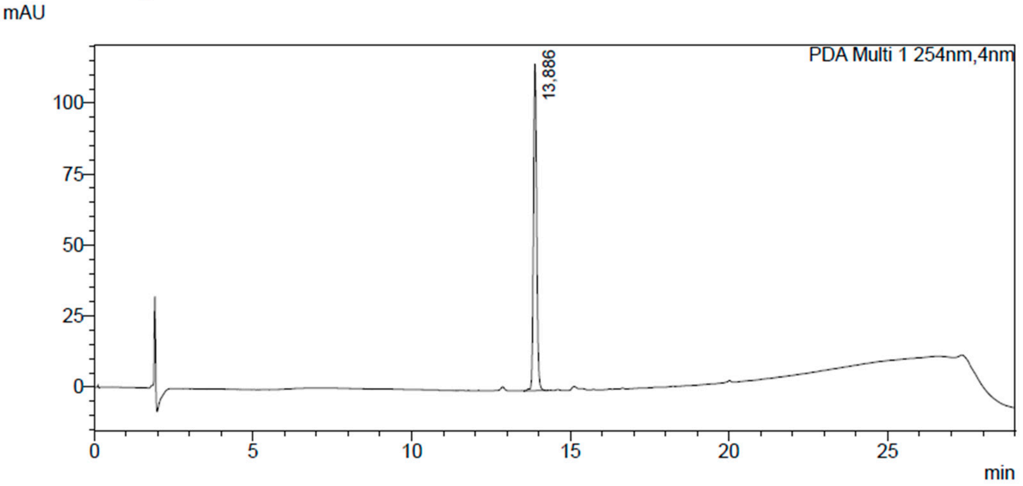

<Peak Table>

PDA Ch1 254nm

Peak#	Ret. Time	Area	Height	Conc.	Unit	Mark	Name
1	13,886	807016	115086	100,000			
Total		807016	115086				

Figure S24. Chromatogram of compound **5**.

<Chromatogram>

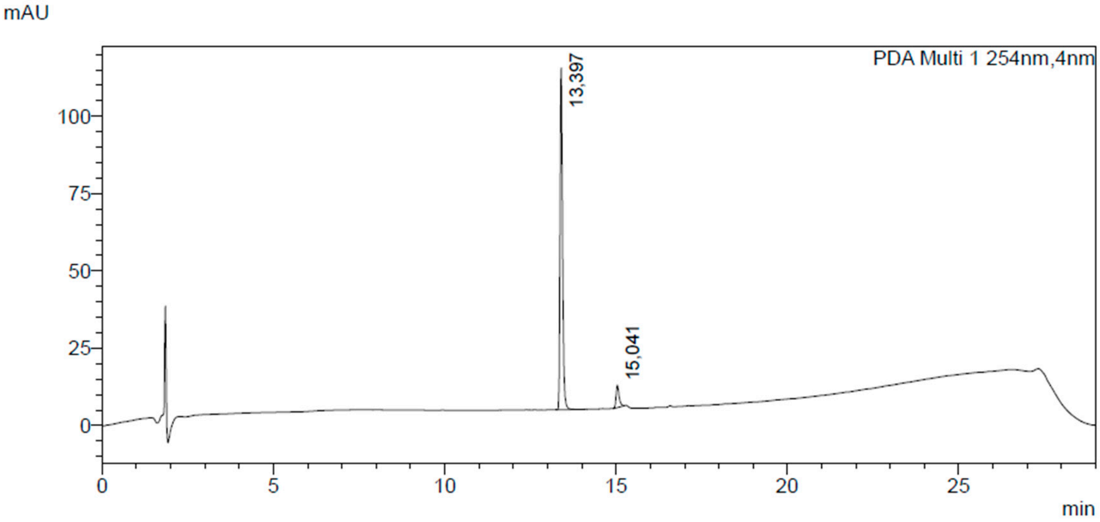

<Peak Table>

PDA Ch1 254nm

Peak#	Ret. Time	Area	Height	Conc.	Unit	Mark	Name
1	13,397	562783	110582	92,635			
2	15,041	44742	7185	7,365			
Total		607525	117767				

Figure S25. Chromatogram of compound **6**.

<Chromatogram>

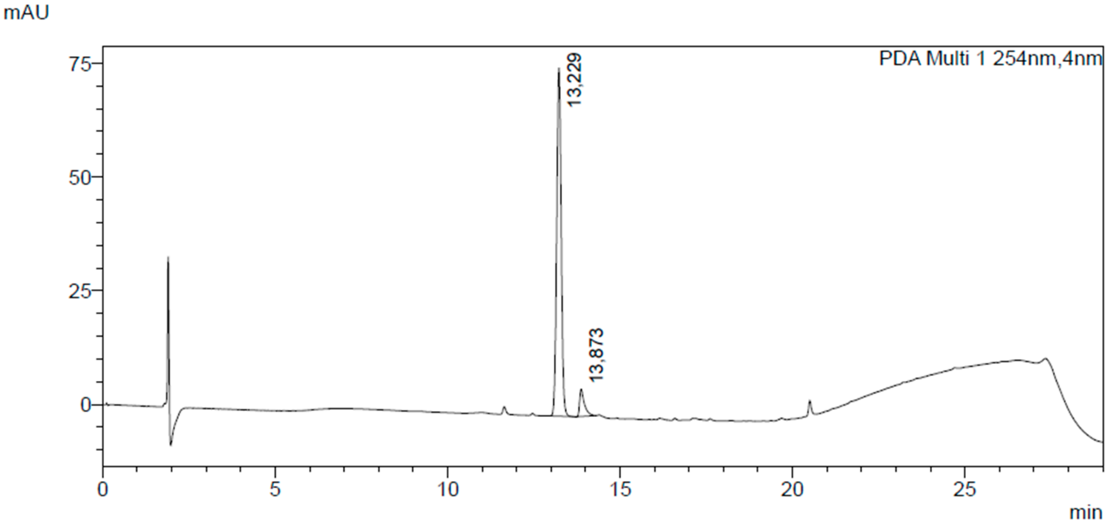

<Peak Table>

PDA Ch1 254nm

Peak#	Ret. Time	Area	Height	Conc.	Unit	Mark	Name
1	13,229	696429	76568	92,347			
2	13,873	57712	5992	7,653			
Total		754141	82560				

Figure S26. Chromatogram of compound **7**.

<Chromatogram>

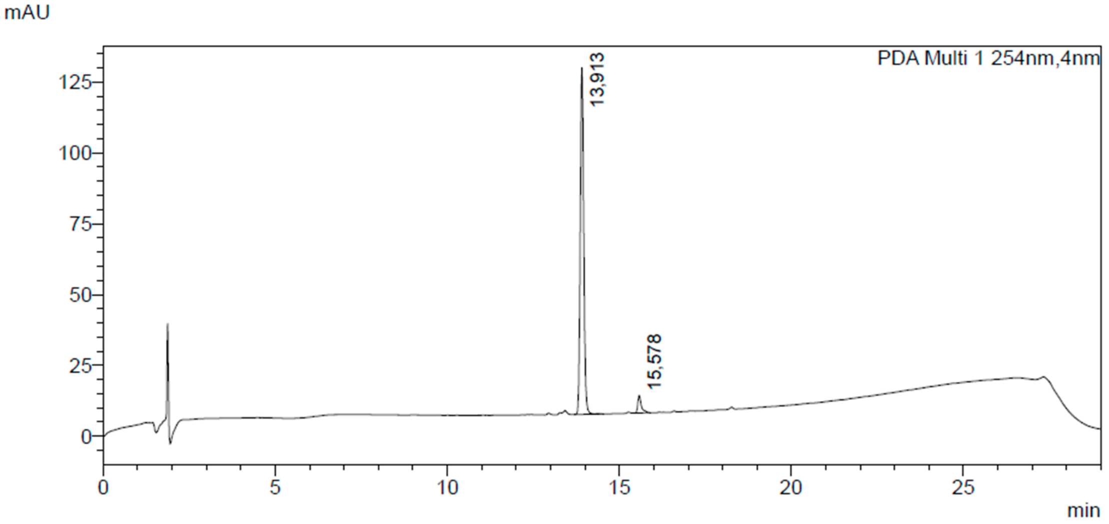

<Peak Table>

PDA Ch1 254nm

Peak#	Ret. Time	Area	Height	Conc.	Unit	Mark	Name
1	13,913	861093	122365	95,113		S	
2	15,578	44246	6156	4,887			
Total		905338	128520				

Figure S27. Chromatogram of compound **8**.

<Chromatogram>

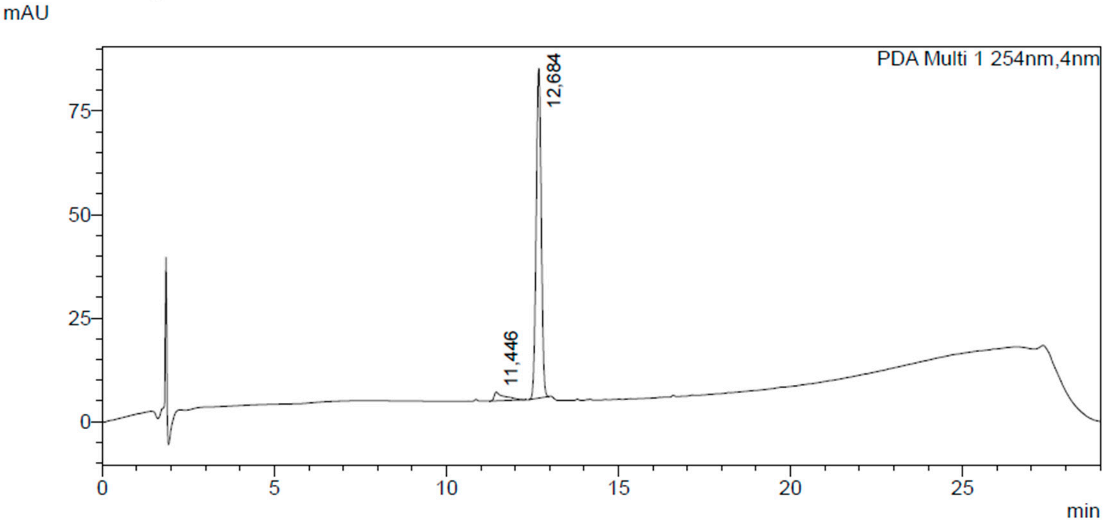

<Peak Table>

PDA Ch1 254nm

Peak#	Ret. Time	Area	Height	Conc.	Unit	Mark	Name
1	11,446	45236	2157	5,281			
2	12,684	811378	79707	94,719			
Total		856614	81864				

Figure S28. Chromatogram of compound **9**.
